# Characterization of Next-Generation Inhibitors for the Inward-Rectifier Potassium Channel K_ir_2.1: Discovery of VU6080824

**DOI:** 10.1021/acsmedchemlett.5c00297

**Published:** 2025-08-29

**Authors:** Renn A. Duncan, Daniel H. Haymer, Roman M. Lazarenko, Liangping Li, Yvette Blackwell, Emily L. Days, Srinivasan Krishnan, Analisa Thompson Gray, Olivier Boutaud, Darren W. Engers, Craig W. Lindsley, Jerod S. Denton, Aaron M. Bender

**Affiliations:** † Warren Center for Neuroscience Drug Discovery, 5718 Vanderbilt University, Nashville, Tennessee 37232, United States; ‡ Department of Pharmacology, Vanderbilt University, Nashville, Tennessee 37232, United States; § Department of Anesthesiology, Vanderbilt University Medical Center, Nashville, Tennessee 37232, United States; ∥ VVanderbilt Institute of Chemical Biology, Vanderbilt University, Nashville, Tennessee 37232, United States; ⊥ Department of Chemistry, Vanderbilt University, Nashville, Tennessee 37240, United States; # Department of Biochemistry, Vanderbilt University, Nashville, Tennessee 37205, United States

**Keywords:** inward-rectifier potassium channel, SAR, thallium flux, manual patch clamp, pharmacokinetics, deuterium

## Abstract

ML133 is a selective inhibitor of the inward-rectifier potassium channel K_ir_2.1 and has found extensive use as a tool with which to probe K_ir_ biology. Despite its utility as a tool compound, ML133 has only modest on-target potency (manual patch clamp (MPC) K_ir_2.1 IC_50_ = 1.5 μM, pH 7.4), and its *in vivo* pharmacokinetics (PK) were previously uncharacterized. In the present study, we report a next-generation series of K_ir_2.1 inhibitors based on the ML133 scaffold, along with the rat PK of ML133 and selected analogs. Compound **5s** (VU6080824) was ultimately identified as having superior potency to ML133 in both the thallium flux and MPC functional assays and has excellent PK properties suitable for use as an improved K_ir_2.1 tool compound in rodents.

## Graphical abstract

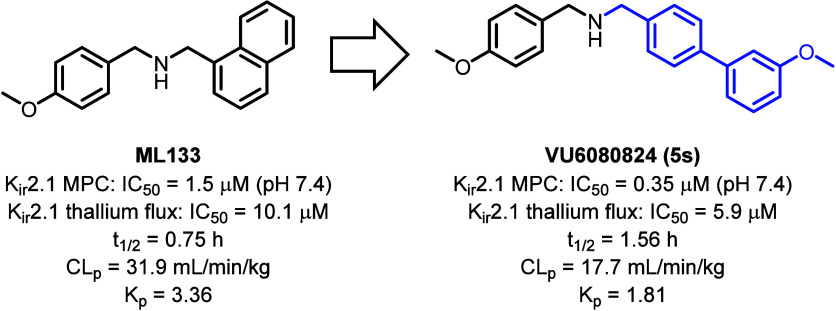

The inward-rectifier potassium channels (K_ir_) play critical roles in diverse biological functions and are characterized by broad tissue distribution. At least 7 subfamilies of K_ir_ channels have been identified (K_ir_1-K_ir_7), and each class is further subdivided into specific isoforms. Most K_ir_ channels exist as either homomeric or heteromeric tetramers arranged around a central pore, through which potassium ions (K^+^) move along an electrochemical gradient. “Inward-rectifier” refers to the tendency of the K_ir_ channels to pass this ionic current predominantly in the inward direction under voltage-clamp conditions.^1^

It is well-established that the K_ir_ channels are valuable therapeutic targets for a wide range of indications including cardiovascular, metabolic, renal, and neurological.^1–5^ Specifically, K_ir_2.1, the second member of the K_ir_ class to be cloned, is broadly expressed in bodily tissues including cardiac cells,^6^ macrophages,^7^ neutrophils,^8^ astrocytes,^9^ and glial cells.^10^ Moreover, K_ir_2.1 mutations have been linked to disorders including periodic paralysis, Anderson-Tawil syndrome (ATS), and short-QT syndrome.^11–13^ Selective modulation of K_ir_2.1, therefore, would be of immense value both for therapeutic development and as a tool to further understand K_ir_2.1 biology.

The high sequence homology of the K_ir_ families within the pore domain, including the K_ir_2 family, has challenged the development of selective tool molecules.^1,2^ In general, the known pharmacological toolbox for the K_ir_ channels contains only weak and/or nonselective isoform inhibitors (although selective inhibitors for certain isoforms, e.g., K_ir_1.1, have been described).^14^ Such tool molecules are often further limited by poor physicochemical properties and lack of “drug-likeness”. Although ion flux-based high-throughput screening (HTS) approaches have found some success in the identification of new chemical matter for various members of the K_ir_ family, many of the available chemotypes require extensive optimization.^1^ In the case of K_ir_2.1 specifically, the available tools are limited to weakly active molecules repurposed from other indications (e.g., tamoxifen, chloroquine, and thiopental),^15–17^ nonspecific divalent cation inhibition (barium, Ba^2+^),^18^ or the HTS-derived semiselective inhibitor ML133.^2^

ML133 (*N*-(4-methoxybenzyl)-1-(naphthalen-1-yl)­methanamine) is a pH-dependent K_ir_2.1 probe identified from a thallium flux HTS campaign in association with the Molecular Libraries Probe Production Centers Network (MLPCN) (Figure 1).^2^ Although ML133 represents an encouraging step toward the identification of a potent small molecule K_ir_ probe (human K_ir_2.1 manual patch clamp (MPC) IC_50_ = 1.8 μM, pH = 7.4 in the original report), the compound is relatively nonselective against the other channels in the K_ir_2.*x* family. Encouragingly, however, ML133 has appreciable selectivity against other K_ir_ channels including K_ir_1.1, K_ir_4.1 and K_ir_7.1.^2^ The on-target potency and selectivity of ML133 has facilitated its use as a probe across many disease models including sour taste transduction,^19^ regulation of neuronal processes,^20^ modulation of autophagy in endothelial progenitor cells,^21^ and traumatic brain injury.^22^

**Figure 1:**
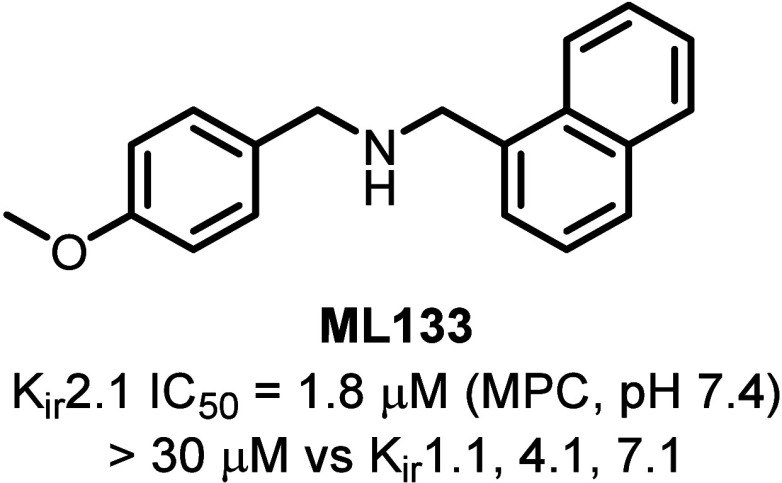
Chemical structure, manual patch clamp (MPC), and selectivity data for ML133.

Given the widespread success of ML133 as a K_ir_2.1-specific molecular probe, we became interested in the identification of next-generation K_ir_2.1 inhibitors based on the ML133 scaffold. The previously reported structure–activity relationship (SAR) data for ML133 were quite steep, and minor modifications to the substituted dibenzylamine scaffold were generally observed to ablate all K_ir_2.1 activity (for example, all examined replacements to the 4-methoxybenzyl motif resulted in inactive compounds).^2^ Additionally, the rodent pharmacokinetics (PK) for ML133 were not known, further limiting its utility as an *in vivo* tool (i.e., understanding appropriate doses for achieving sufficient plasma exposure, correlation of PK with any observed behavioral phenotypes in animal studies). Toward the identification of next-generation, ML133-based K_ir_2.1 inhibitors, our goals for the present study were therefore 2-fold: (1) an expanded and comprehensive SAR campaign, examining all facets of the ML133 scaffold, to identify molecules with improved K_ir_2.1 inhibition, and (2) PK characterization for ML133 and related analogs in rats to further enable *in vivo* work, as the MLPCN did not allow for in-depth DMPK profiling at the time.

We initially sought to further explore replacements to the 4-methoxybenzyl group, and a series of molecules were synthesized in which the 1-naphthyl pendant was held constant in the context of 4-methoxybenzyl replacements. Analogs in this series were prepared via a simple reductive amination reaction between 1-naphthaldehyde (**2**) and the corresponding amine (Scheme 1).

**Scheme 1:**
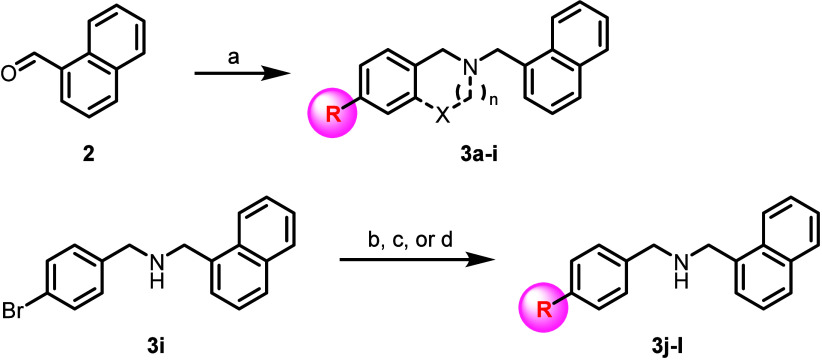
Synthesis of Compounds **3a**-**l** ^a^

a Reagents and conditions: (a) 4-substituted benzylamine, NaBH­(OAc)_3_, DCM, r.t., 4–80% (**3a**–**i**). (b) 2-Methoxyethanol, Cs_2_CO_3_, RockPhos-Pd-G3, toluene, 100 °C, 30% (**3j**). (c) Isothiazolidine 1,1-dioxide, K_3_PO_4_, [Pd­(allyl)­(*t*-BuBrettPhos)]­OTf,^24^ *t*-BuOH, 110 °C, 11% (**3k**). (d) Dimethylphosphine oxide, K_3_PO_4_, Pd_2_(dba)_3_, XantPhos, 1,4-dioxane, 110 °C, 36% (**3l**).

In the original SAR report, all modifications to the 4-methoxybenzyl moiety were found to ablate K_ir_2.1 activity, and selected examples were reported (e.g., 4-Cl and 4-OCF_3_). With an understanding of this steep SAR, we therefore decided to begin by profiling specific hydrogen bond acceptors as methoxy replacements, hoping to more closely mimic the polarity and hydrogen-bonding characteristics of the 4-OMe group. Initially, a diverse group of hydrogen bond acceptor motifs were surveyed at the 4-phenyl position (exemplified by **3a**–**e**, Table 1). Unfortunately, as was observed in the original SAR campaign, all attempted modifications/replacements to the methoxy substituent were unfruitful. In some cases, weaker activity (> 10 μM, with diminished % efficacy) was observed (as in the case of difluoromethoxy analog **3a**, nitrile **3b**, and pyrazole **3e**). Cyclization of the left-hand pendant (**3g** and **3h**, prepared via analogous reductive amination chemistry) was also detrimental to K_ir_2.1 potency, as was the introduction of a hydrogen bond donor (primary alcohol **3f**). To access a greater diversity of 4-substitutions, additional O-, N-, and P-linked analogs were prepared via transition metal cross-coupling chemistry starting from 4-bromo intermediate **3i** (exemplified by **3j**–**l**).^23–25^ For cross-couplings involving challenging nucleophiles (e.g., sultam **3k**), a π-allylpalladium precatalyst derived from *t*BuBrettPhos ([Pd­(allyl)­(*t*BuBrettPhos)]­OTf, prepared as previously described)^24^ proved particularly useful (Scheme 1). Unfortunately, all examined replacements to the 4-methoxy group continued to dramatically diminish K_ir_2.1 activity. These results further corroborate the necessity of the 4-methoxy substitution for maintaining K_ir_2.1 inhibition within this chemotype.

**Table 1:**
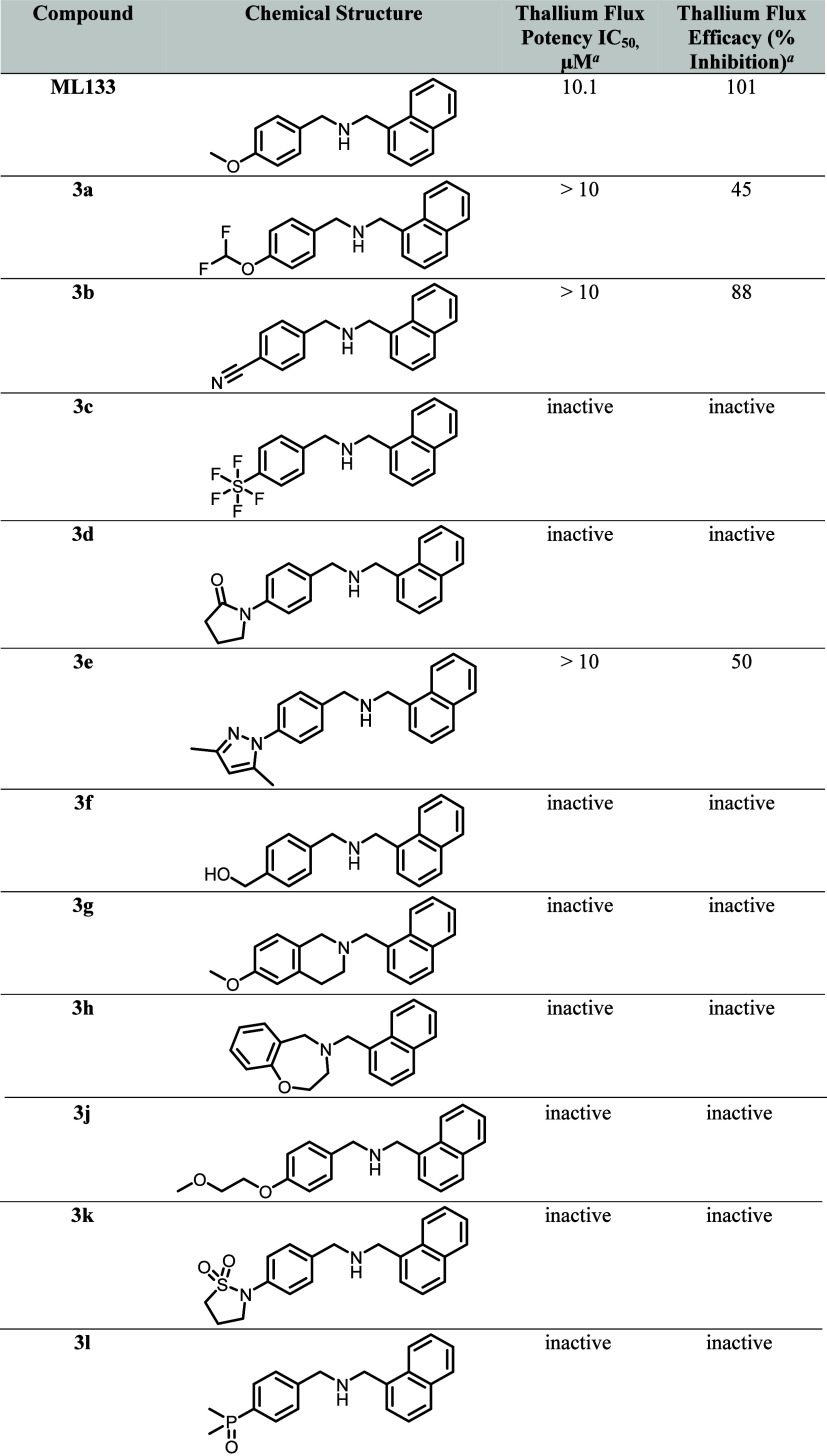
K_ir_2.1 Thallium Flux Potency and Efficacy Data for ML133, Compounds **3a**-**h**, **3j**-**l**

a Live cells expressing human K_ir_2.1 were stimulated with thallium solution and measured in real time for changes in fluorescent signal (482ex, 536em). The slopes of the change in fluorescence were fit to a four-parameter logistic equation to estimate the reported IC_50_ (μM) and efficacy (% inhibition). Test compounds that did not fit the 4PL model within the concentration range tested (> 10 μM) have the 30 μM measured value shown expressed relative to 30 μM ML133 (% inhibition). Values represent a single experiment tested in triplicate. ML133 estimated IC_50_ 10.1 μM (pIC_50_ 4.98 ± 0.05 SEM) is the mean reported over 9 experimental plates in triplicate representing dates of the test compounds included in the table.

Next, we turned our attention to replacements to the 1-naphthyl pendant (Scheme 2). Starting from (4-methoxyphenyl)­methanamine (**4**), analogs **5a**–**i** were prepared via reductive amination chemistry, and analogs **5j** and **5k** were accessed via substitution chemistry from 4-methoxybenzyl chloride (**6**). Analogously to Scheme 1, a greater diversity of substitutions could be accessed utilizing cross-coupling chemistry. Specifically, Suzuki-Miyaura chemistry starting from aryl bromide intermediate **5i** afforded analogs **5m**–**u**; to avoid a historically challenging coupling with a 2-pyridyl boron reagent,^26^ aryl bromide **5i** was converted to the pinacol boronate prior to coupling with 2-bromopyridine to give **5l**. Buchwald-Hartwig cross-couplings smoothy furnished analogs **5v-z**. The SAR results for **5a**–**h**, **5j**–**z** are outlined in Table 2. In short, this exercise was found to be much more fruitful; after synthesizing > 50 novel ML133 analogs in this context, several were found to have comparable or improved thallium flux potency relative to ML133 (Table 2).

**Scheme 2:**
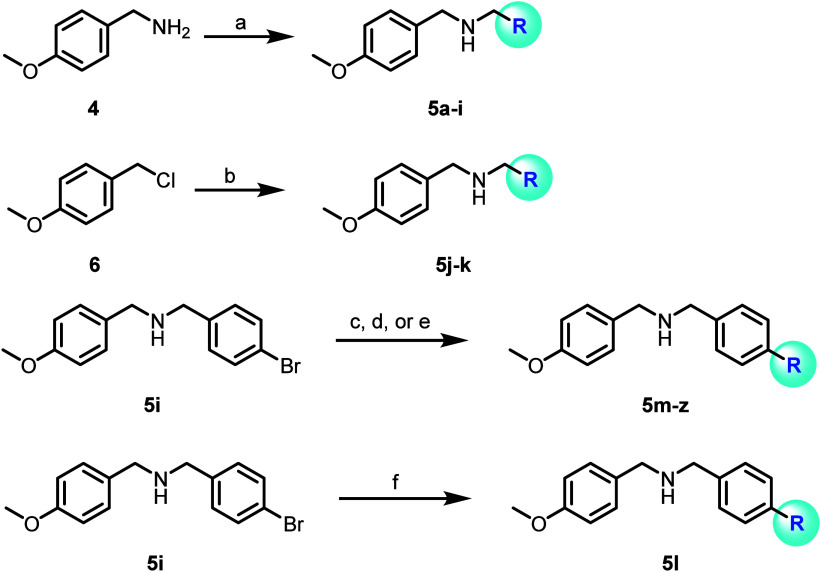
Synthesis of Compounds **5a**-**z** ^a^

a Reagents and conditions: (a) substituted aldehyde, NaBH­(OAc)_3_, DCM, r.t., 28–70% (**5a**–**i**). (b) Substituted amine, K_2_CO_3_, MeCN, r.t., 7–25% (**5j**–**k**). (c) Substituted boronic acid or ester, Cs_2_CO_3_, Pd­(dppf)­Cl_2_, 1,4-dioxane, H_2_O, 100 °C, 41–66% (**5m**–**u**). (d) Substituted amine, Cs_2_CO_3_, Pd_2_(dba)_3_, RuPhos, 1,4-dioxane, 100 °C, 66–83% (**5v**–**y**). (e) 1,2-Thiazinane 1,1-dioxide, K_3_PO_4_, [Pd­(allyl)­(*t*-BuBrettPhos)]­OTf,^24^ 1,4-dioxane, 100 °C, 42% (**5z**). (f) (i) Bis­(pinacolato)­diboron, potassium acetate, Pd­(dppf)­Cl_2_, 1,4-dioxane, 100 °C; (ii) 2-bromopyridine, Cs_2_CO_3_, Pd­(dppf)­Cl_2_, 1,4-dioxane, H_2_O, 100 °C, 16% (**5l**).

**Table 2:**
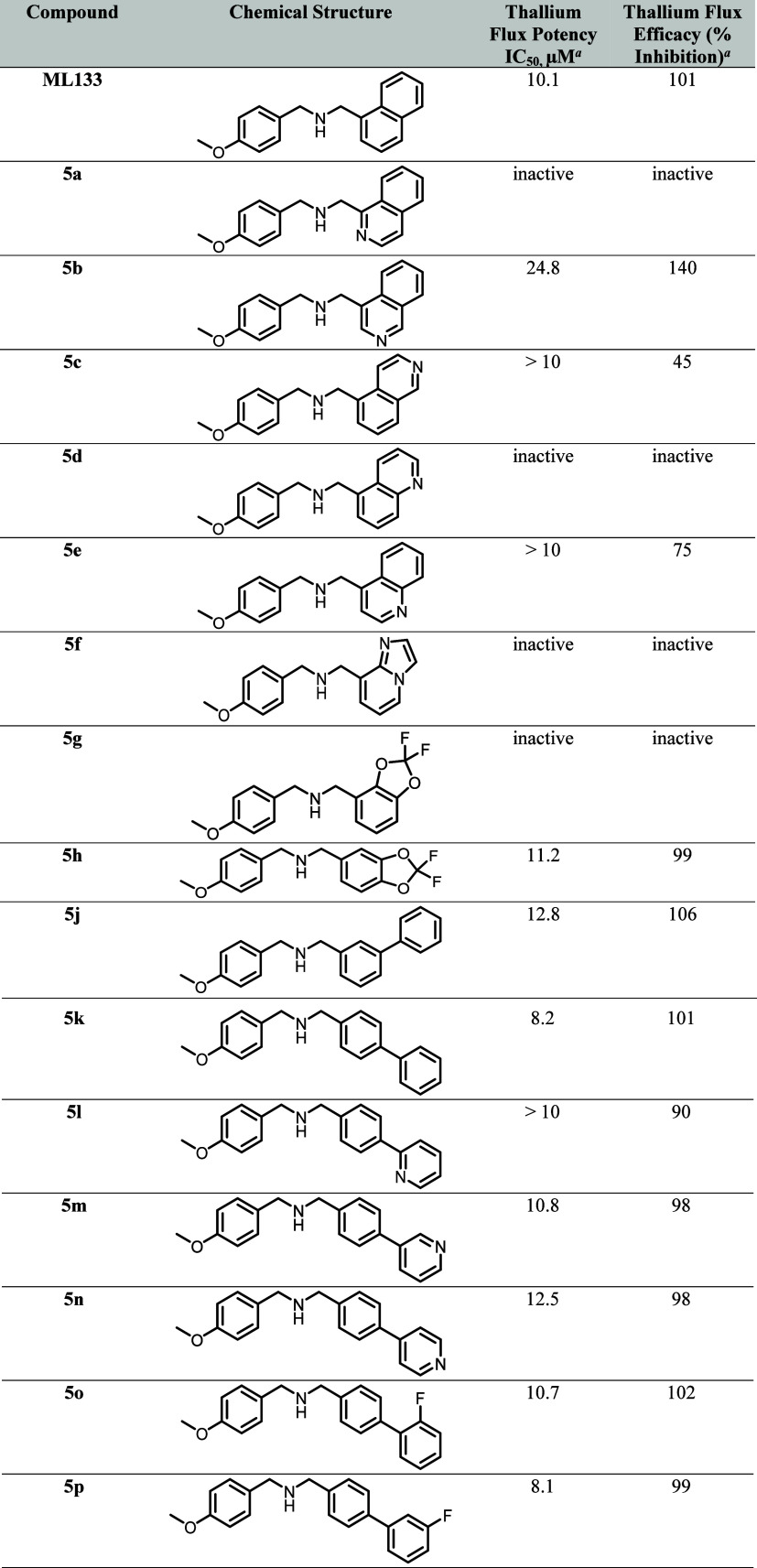

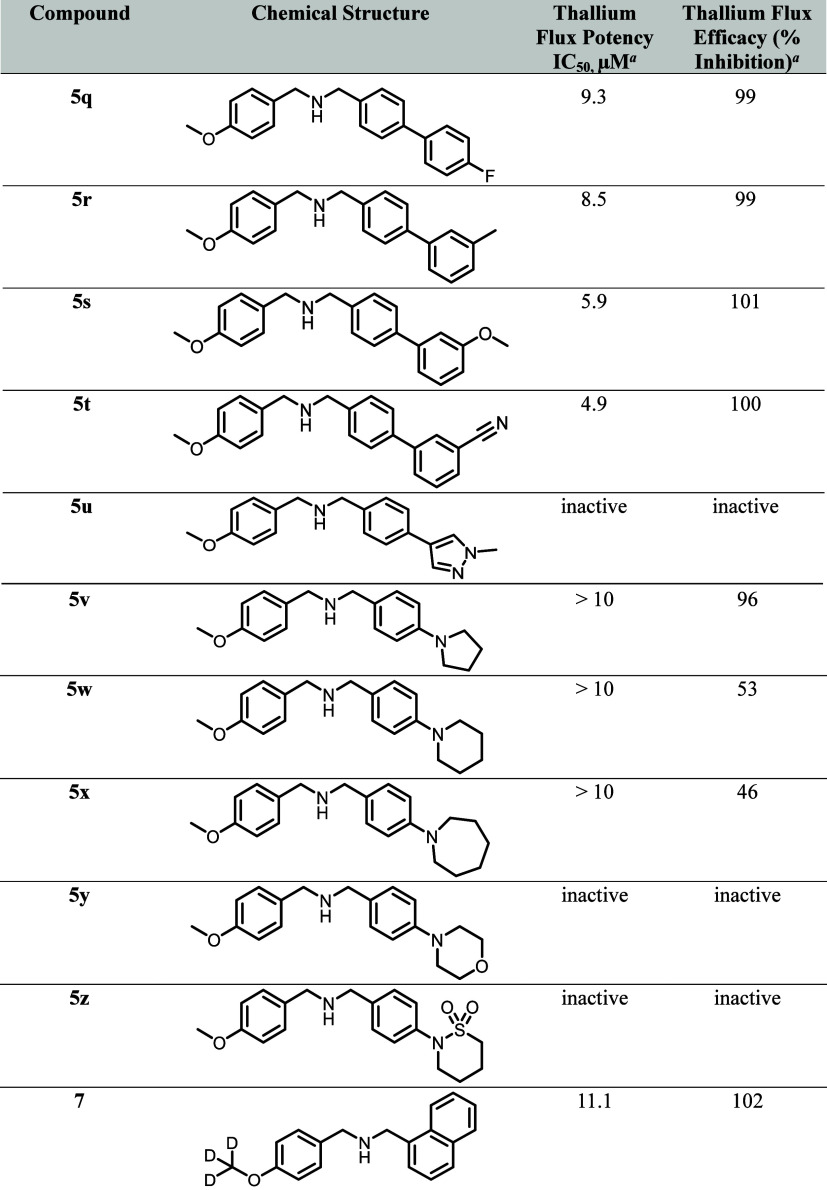
K_ir_2.1 Thallium Flux Potency and Efficacy Data for ML133, Compounds **5a**-**h**, **5j**-**z**, **7**

a Live cells expressing human K_ir_2.1 were stimulated with thallium solution and measured in real time for changes in fluorescent signal (482ex, 536em). The slopes of the change in fluorescence were fit to a four-parameter logistic equation to estimate the reported IC_50_ (μM) and efficacy (% inhibition). Test compounds that did not fit the 4PL model within the concentration range tested (> 10 μM) have the 30 μM measured value shown expressed relative to 30 μM ML133 (% inhibition). Values represent a single experiment tested in triplicate. ML133 estimated IC_50_ 10.1 μM (pIC_50_ 4.98 ± 0.05 SEM) is the mean reported over 9 experimental plates in triplicate representing dates of the test compounds included in the table.

An *aza*-scan of the 1-naphthyl group largely resulted in compounds with attenuated activity relative to ML133, although isoquinoline **5b**, while approximately 2-fold less potent than ML133 in the thallium flux assay, was found to be highly efficacious (140% inhibition). Alternative 6/5 bicyclic replacements (e.g., imidazopyridine **5f** and benzo­[*d*]­[1,3]­dioxole **5g**) were inactive, although, interestingly, regioisomeric benzo­[*d*]­[1,3]­dioxole **5h** was found to be roughly equipotent to ML133. 1-([1,1’-Biphenyl]-3-yl) and 1-([1,1’-biphenyl]-4-yl) replacements (**5j** and **5k**) were also tolerated, particularly 1-([1,1’-biphenyl]-4-yl) analog **5k** (thallium flux IC_50_ = 8.2 μM). Accordingly, we next held the 1-([1,1’-biphenyl]-4-yl) motif constant, and surveyed systematic substitutions at the *ortho*, *meta*, and *para* positions (*aza*: **5l**–**5n**; and fluoro: **5o**–**5q**). In both series, substitution at the *meta* position afforded analogs with the greatest potencies (3-pyridine **5m** and 3-fluorophenyl **5p**). Additional *meta* substitutions (3-methyl **5r**, 3-methoxy **5s** and 3-cyano **5t**) were also well tolerated and indeed afforded the most potent K_ir_2.1 inhibitors within this chemotype to date (thallium flux IC_50_ *s* < 6 μM for **5s** and **5t**). Substitution with alternative heteroaromatics at the 4-phenyl position were not tolerated (e.g., pyrazole **5u**). Within the *N*-linked series (**5v**–**5z**), prepared via Buchwald-Hartwig chemistry, all examined compounds were either inactive or had attenuated activity compared to ML133, apart from pyrrolidine **5v** (see Table 2).

*O*–Demethylation of aryl methoxy groups is a common metabolic degradation pathway,^27,28^ and, in parallel, we were interested in the design of an ML133 analog that might circumvent this potential biotransformation. Accordingly, we also synthesized the direct OCD_3_ analog of ML133 (**7**, Scheme 3). In the thallium flux assay, **7** was found to be approximately equipotent to ML133 (see Table 2).

**Scheme 3:**
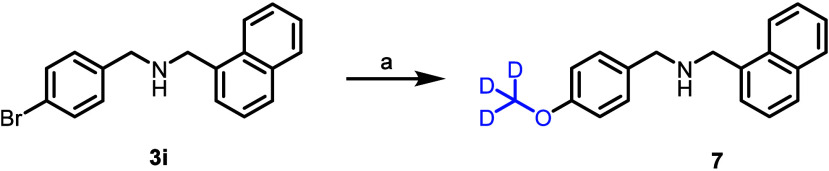
Synthesis of Compound 7^a^

a Reagents and conditions: (a) methanol-*d*4, Cs_2_CO_3_, RockPhos-Pd-G3, toluene, 100 °C, 42%.

Selected novel analogs were further profiled relative to ML133 in the manual patch clamp (MPC) assay. In the original ML133 report, the compound was found to have 1.8 μM potency at pH 7.4 (ML133 is a pH-dependent probe due to the presence of the basic amine).^2^ Upon retesting, ML133 was found to have a potency value of 1.51 μM, in good agreement with the initial publication. Selected next-generation analogs with comparable or improved potency in the thallium flux assay were chosen for MPC profiling (Table 3). Interestingly, OCD_3_ analog **7** was approximately 3–4-fold less potent than ML133 in this context (as was benzo­[*d*]­[1,3]­dioxole **5h**). 1-([1,1’-Biphenyl]-4-yl) analog **5k** was equipotent relative to ML133. Encouragingly, *meta*-substituted 1-([1,1’-biphenyl]-4-yl) analogs **5j**, **5p**, **5s** and **5t** were all ∼ 3–5-fold more potent than ML133 in MPC; these analogs are therefore the most potent K_ir_2.1 analogs yet described for the dibenzylamine chemotype.

**Table 3:** Manual Patch Clamp Data for Selected K_ir_2.1 Inhibitors^a^

Compound	IC_50_, μM	Emax,%
**ML133**	1.51^b^	99
**5h**	3.03	92
**5j**	0.39	100
**5k**	1.39	99
**5p**	0.30	98
**5s**	0.35	100
**5t**	0.48^c^	98
**7**	3.94	82

a Electrophysiological characterization of K_ir_2.1 current inhibition. Responses were measured at −120 mV and normalized to the 2 mM Ba^2+^ full block. IC_50_ values were derived from a four-parameter logistic fit. *E*_max_ indicates the % inhibition of Ba^2+^-sensitive current at the highest tested concentration (3–30 μM). MPC experiments were run at pH 7.4 with a top compound concentration of 10 μM, unless noted.

b 30 μM top concentration.

c 3 μM top concentration.

Differences in compound potency between the thallium flux and MPC assays are routinely observed, with MPC considered the “gold standard” measurement (in MPC, voltage clamp electrophysiology directly measures the potassium current at specific voltages and channel open states, rather than a nonphysiological cation (e.g., thallium).^29–31^ The potency difference observed for deuterated compound **7** relative to ML133 in this context is noteworthy and in line with the steep SAR observed for the attempted replacements to the 4-methoxy group. Further study, however, will be necessary to refine the true extent to which this hydrogen/deuterium exchange affects the MPC potency, and to understand any mechanism(s) behind this observation.

While ML133 has found use as a K_ir_2.1 tool molecule across many disease models, the *in vivo* PK of this and related compounds within the dibenzylamine chemotype have not previously been characterized. As a highly efficient method for profiling the PK of a compound set, we therefore examined ML133 (alongside 9 additional next-generation K_ir_2.1 inhibitors) in our rat PK PBL cassette dosing platform (Table 4).^32–34^ From this experimental setup, administration of a 4-compound bolus dose, plus a standard control, can be used to determine a variety of PK parameters in a high-throughput fashion. Specifically, Sprague–Dawley (SD) rats were given 0.2 mg/kg of each K_ir_2.1 inhibitor (i.v. dosing, 4 compounds +1 control, total dose 1 mg/kg) with PK measured across set time points up to 24 h. Additionally, a separate cohort of animals was utilized to assess total plasma-brain levels (PBL, ratio reported as K_p_) of compound at a 0.25 time point. In this fashion, standard plasma-based PK parameters (elimination *t*_1/2_, mean resonance time (MRT), plasma clearance (CL_p_), and volume of distribution (V_ss_)) were measured alongside a parallel assessment of total brain exposure.

**Table 4:** Rat PK PBL Cassette Data for Selected K_ir_2.1 Inhibitors^a^

Compound	Elim. *t*_1/2_ (h)	MRT (h)	CL_p_ (mL/min/kg)	V_ss_ (L/kg)	AUC (h*ng/mL)	K_p_
**ML133**	0.75	0.78	31.9	1.50	104	3.36
**7**	1.39	1.24	22.8	1.70	146	3.30
**5b**	0.29	0.36	18.2	0.39	183	1.05
**5h**	1.33	0.87	30.4	1.58	110	2.13
**5j**	1.18	1.00	31.7	1.90	105	4.23
**5k**	1.68	1.08	22.6	1.46	148	1.82
**5p**	0.69	0.76	30.6	1.39	109	2.27
**5r**	1.73	1.31	20.6	1.62	162	2.29
**5s**	1.56	0.99	17.7	1.06	189	1.81
**5t**	1.66	0.99	20.2	1.20	165	1.25

a Compounds are administered to male SD rats (*n* = 1) as a 0.2 mg/kg i.v. dose (8% EtOH, 33% PEG400, 58% DMSO (0.5 mL/kg)).

In general, compounds within this chemotype were found to have short to moderate *t*_1/2_s (0.29 – 1.73 h) and MRTs (0.36 – 1.31 h), with CL_p_ values in the low to moderate range (17.7 – 31.9 mL/min/kg). All profiled compounds were also found to have a moderate V_ss_ (0.39 – 1.90 L/kg), and all were brain penetrant (K_p_ *s* ≥ 1.05). Interestingly, OCD_3_ analog **7** (VU6079685) was found to have a ∼ 2-fold longer *t*_1/2_ compared to ML133, and a ∼ 1.4-fold lower overall plasma clearance. These data indicate that *O*-demethylation may contribute to the metabolic clearance of this chemotype, an effect that can be readily attenuated through deuterium incorporation (kinetic isotope effect).^35,36^

Although several compounds in this series proved attractive with respect to overall PK, analog **5s** (VU6080824) was particularly encouraging. This compound displayed low plasma clearance (CL_p_ = 17.7 mL/min/kg), with a *t*_1/2_ of 1.56 h. Additionally, VU6080824 was found to be robustly brain penetrant (K_p_ = 1.81). These PK data, alongside the improved MPC potency of this compound relative to ML133 (IC_50_ = 0.35 μM, pH 7.4), highlight VU6080824 as a next-generation K_ir_2.1 inhibitor with superior potency and PK properties relative to ML133.

In the thallium flux assay, VU6080824 was found to be selective relative to K_ir_1.1 and K_ir_4.1 up to 10 μM (K_ir_1.1 and K_ir_4.1 IC_50_ *s* > 10 μM), although additional refinement will be necessary to understand the true selectivity ratios for each given the relatively modest potency of VU6080824 in the K_ir_2.1 thallium flux assay (IC_50_ = 5.9 μM). With respect to selectivity against other members of the K_ir_2.*x* family, VU6080824 was found to be approximately 2-fold selective relative to K_ir_2.2 and K_ir_2.3, as measured by MPC (IC_50_s = 0.75 μM and 0.86 μM, respectively). These data are in line with the overall selectivity profile observed for ML133,^2^ and indicate that further structural refinements will be necessary to improve selectivity within the Kir2.*x* family.

Given the favorable PK results observed for OCD_3_ analog **7** relative to ML133, the three possible OCD_3_ analogs of VU6080824 (**5s**) were also prepared in an analogous fashion (left-hand pendant OCD_3_, right-hand pendant OCD_3_, left–right dual OCD_3_; see the Supporting Information for further details). In this context, however, none of the deuterated analogs were superior to **5s** with respect to PK, and all showed comparable (if slightly inferior) clearance profiles. Additionally, all three OCD_3_ analogs displayed comparable K_ir_2.1 potencies in both the thallium flux (4.3 – 5.4 μM) and MPC assays (0.21 – 0.33 μM) to **5s**, and similar selectivity profiles relative to K_ir_2.2 and K_ir_2.3 (generally 1–3 fold selectivity; see the Supporting Information for full potency and PK details). The effects of deuterium incorporation within this chemotype thus appear to be highly context dependent.

In conclusion, we have profiled a novel set of K_ir_2.1 inhibitors, using the substituted dibenzylamine ML133 chemotype as a scaffold for chemical diversification. From this exercise, substitutions on the 1-naphthyl pendant identified next-generation analogs with improved potency relative to ML133 in both the thallium flux and MPC functional assays. Selected compounds, along with ML133, were further profiled in rat PK PBL cassettes to understand the *in vivo* properties of this series. VU6080824 (1-(3′-Methoxy-[1,1’-biphenyl]-4-yl)-*N*-(4-methoxybenzyl)­methanamine) ultimately emerged as a superior K_ir_2.1 tool molecule, which we hope will find widespread use as a probe for K_ir_ biology.

**Safety Statement.** No unexpected or unusually high safety hazards were encountered.

## Supplementary Material



## Notes

Experimental methods for compound synthesis and characterization, and experimental protocols for molecular pharmacology (thallium flux and manual patch clamp functional assays). Experimental methods for PK cassette studies (PDF)

## Abbreviations

ATS: Anderson-Tawil Syndrome
AUC: area under the curve
CL_p_: plasma clearance (mL/min/kg)
DCM: dichloromethane
GPCR: G protein-coupled receptor
HTS: high-throughput screen
K_ir_: inward-rectifier potassium channel
K_p_: total brain to plasma ratio
MeCN: acetonitrile
ML133: (*N*-(4-methoxybenzyl)-1-(naphthalen-1-yl)­methanamine)
MLPCN: Molecular Libraries Probe Production Centers Network
MPC: manual patch clamp
MRT: mean resonance time (h)
PBL: plasma-brain levels
PK: pharmacokinetics
SAR: structure–activity relationship
SD: Sprague–Dawley rats
*t*_1/2_: half-life (h)
*t*-BuOH: *tert*-butanol
V_ss_: volume of distribution (steady state, L/kg)
