# Supplementary material for: Characterization of Next-Generation Inhibitors for the Inward-Rectifier Potassium Channel Kir2.1: Discovery of VU6080824
Source: ACS Med Chem Lett. 2025 Aug 29;16(9):1762–71. doi: 10.1021/acsmedchemlett.5c00297 (PMC12434521; doi:10.1021/acsmedchemlett.5c00297)
Supplement: Supplementary file 1 [file ml5c00297_si_001.pdf]

Supporting Information

Characterization of Next-Generation Inhibitors for the Inward-Rectifier Potassium Channel K_{ir}2.1: Discovery of VU6080824

Renn A. Duncan,[a,b,^] Daniel H. Haymer,[a,b,^] Roman M. Lazarenko,[c] Liangping Li,[d] Yvette Blackwell,[c] Emily L. Days,[d] Srinivasan Krishnan,[a,b] Analisa Thompson Gray,[a,b] Olivier Boutaud,[a,b] Darren W. Engers,[a,b] Craig W. Lindsley,[a,b,e,f,*] Jerod S. Denton,[b,c,d,*] Aaron M. Bender[a,b,*]

a. Warren Center for Neuroscience Drug Discovery, Vanderbilt University, Nashville, Tennessee 37232, United States
b. Department of Pharmacology, Vanderbilt University, Nashville, Tennessee 37232, United States
c. Department of Anesthesiology, Vanderbilt University Medical Center, Nashville, TN 37232
d. Vanderbilt Institute of Chemical Biology, Vanderbilt University, Nashville, TN 37232
e. Department of Chemistry, Vanderbilt University, Nashville, Tennessee 37240, United States
f. Department of Biochemistry, Vanderbilt University, Nashville, Tennessee 37205, United States

Corresponding author emails: *craig.lindsley@vanderbilt.edu, jerod.s.denton@vumc.org, aaron.bender@vanderbilt.edu

General Experimental Procedures **S2**

Synthesis of Intermediates and Final Analogs **S2**

Characterization of Deuterated Analogs of 5s (9, 11, 13) **S19**

Thallium Flux Assay Experimental Conditions **S22**

Manual Patch Claim Assay Experimental Conditions **S23**

PK PBL Cassette Experimental Conditions **S24**

General Experimental Procedures

All reactions were carried out employing standard chemical techniques. Solvents used for reactions and extraction were ACS grade, and HPLC grade solvents were used for purification. All reagents were purchased from commercial sources and were used without further purification.

All NMR spectra were recorded on a 400 MHz Bruker AV-400 instrument. ^{1}H chemical shifts are reported as δ values in ppm relative to the residual solvent peak (DMSO-d_6 = 2.50). Data are reported as follows: chemical shift, multiplicity (br = broad, s = singlet, d = doublet, t = triplet, q = quartet, p = pentet, dd = doublet of doublets, ddd = doublet of doublet of doublets, td = triplet of doublets, m = multiplet), coupling constant, and integration.

LCMS data were obtained on a Waters QDa (Performance) SQ MS with ESI source. MS parameters were as follows: cone voltage: 15 V, capillary voltage: 0.8 kV, probe temperature: 600° C. Samples were introduced via an Acquity I-Class PLUS UPLC comprised of a BSM, FLSM, CH-A, and PDA. UV absorption was generally observed at 215 nm and 254 nm; 4 nm bandwidth. Column: Phenomenex EVO C18, 1.0 x 50 mm, 1.7 um. Column temperature: 55° C. Flow rate: 0.4 mL/min. Default gradient: 5% to 95% CH_3CN (0.05% TFA) in water (0.05% TFA) over 1.4 min, hold at 95% CH_3CN for 0.1 min.

High resolution mass spectra were obtained on an Agilent 6540 UHD Q-TOF with ESI source. MS parameters were as follows: fragmentor: 150, capillary voltage: 3500 V, nebulizer pressure: 60 psig, drying gas flow: 13 L/min, drying gas temperature: 275° C. Samples were introduced via an Agilent 1290 UHPLC comprised of a G4220A binary pump, G4226A ALS, G1316C TCC, and G4212A DAD with ULD flow cell. UV absorption was observed at 215 nm and 254 nm with a 4 nm bandwidth. Column: Agilent Zorbax Extend C18, 1.8 μm, 2.1 x 50 mm. Gradient conditions: 5% to 95% MeCN in water (0.1% formic acid) over 1 min, hold at 95% MeCN for 0.1 min, 0.5 mL/min, 40° C.

RP-HPLC purifications were performed on a Gilson preparative reversed-phase HPLC system comprised of a 333 aqueous pump with solvent-selection valve, 334 organic pump, GX-271 or GX-281 liquid hander, two column switching valves, and a 155 UV detector. UV wavelength for fraction collection was user-defined, with absorbance generally monitored at 220 nm. Column: Phenomenex Axia-packed Gemini C18, 30 x 50 mm or 30 x 100 mm, 5 μm. Mobile phase: MeCN in H_2O (0.1% TFA) or MeCN in H_2O (0.05% v/v NH_4OH). Gradient conditions: 0.75 min equilibration, followed by user-defined gradient (starting organic percentage, ending organic percentage, duration), hold at 95% MeCN for 1 min, 50 mL/min, 23° C. All tested compounds were ≥ 95% purity as assessed by LCMS and ^{1}H NMR analysis.

Automated flash column chromatography was performed on a Biotage Isolera 1 or a Teledyne ISCO CombiFlash system.

General Procedure A: Reductive Amination for the Synthesis of 3a-i

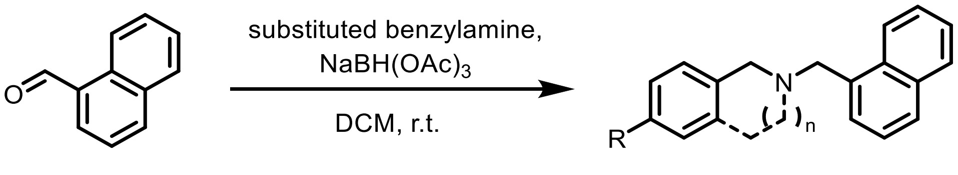

To a stirring solution of 1-naphthaldehyde (1 equiv) and substituted benzylamine (1.5 equiv) in DCM (1 mL) was added sodium triacetoxyborohydride (2 equiv). The resulting reaction mixture was stirred at r.t. overnight or until completion, after which time sat. $NaHCO_3$ solution was slowly added. The aqueous layer was extracted with DCM, and combined organic extracts were

filtered through a hydrophobic phase separator and concentrated. Crude residue was purified by RP-HPLC to give the title compounds.

For acidic purifications (0.1% TFA), fractions containing product were basified with sat. $NaHCO_3$ solution and extracted with DCM. Combined organic extracts were filtered through a hydrophobic phase separator and concentrated to give the title compounds. For basic purifications (0.05% v/v NH_4OH), fractions containing product were extracted with DCM. Combined organic extracts were filtered through a hydrophobic phase separator and concentrated to give the title compounds. Alternatively, for basic purifications, fractions containing product were concentrated directly from the aqueous to give the title compounds.

3a (*N*-(4-(Difluoromethoxy)benzyl)-1-(naphthalen-1-yl)methanamine)

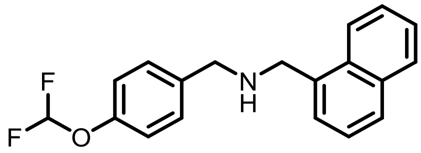

Followed General Procedure A with 1-naphthaldehyde (10 mg, 0.064 mmol, 1 equiv) and (4-(difluoromethoxy)phenyl)methanamine (17 mg, 0.096 mmol, 1.5 equiv) to give the title compound as a white solid after purification by RP-HPLC (12-42% MeCN in 0.1% aqueous TFA solution over 10 min) (0.9 mg, 4%). ^1H NMR (400 MHz, DMSO-d_6) δ 8.14 – 8.12 (m, 1H), 7.93 – 7.89 (m, 1H), 7.81 (d, J = 8.0 Hz, 1H), 7.54 – 7.41 (m, 6H), 7.20 (t, J = 74.3 Hz, 1H), 7.15 – 7.11 (m, 2H), 4.11 (s, 2H), 3.79 (s, 2H). HRMS (TOF, ES+), $C_{19}H_{18}F_2NO$ $[M+H]^+$ calc. mass 314.1351, found 314.1354.

3b (4-(((Naphthalen-1-ylmethyl)amino)methyl)benzonitrile)

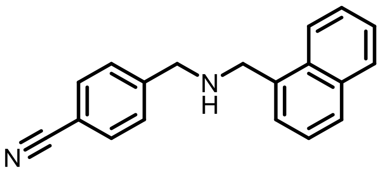

Followed General Procedure A with 1-naphthaldehyde (10 mg, 0.064 mmol, 1 equiv) and 4-(aminomethyl)benzonitrile (13 mg, 0.096 mmol, 1.5 equiv) to give the title compound as a colorless oil after purification by RP-HPLC (27-57% MeCN in 0.05% aqueous NH_4OH solution over 5 min) (2.0 mg, 11%). ^1H NMR (400 MHz, DMSO-d_6) δ 8.18 – 8.12 (m, 1H), 7.94 – 7.90 (m, 1H), 7.84 – 7.79 (m, 3H), 7.62 – 7.58 (m, 2H), 7.55 – 7.44 (m, 4H), 4.16 (s, 2H), 3.93 (s, 2H). HRMS (TOF, ES+), $C_{19}H_{17}N_2$ $[M+H]^+$ calc. mass 273.1386, found 273.1386.

3c (1-(Naphthalen-1-yl)-*N*-(4-(pentafluoro- λ^6-sulfaneyl)benzyl)methanamine)

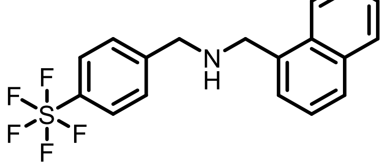

Followed General Procedure A with 1-naphthaldehyde (10 mg, 0.064 mmol, 1 equiv) and (4-(pentafluoro- λ^6-sulfaneyl)phenyl)methanamine (22 mg, 0.096 mmol, 1.5 equiv) to give the title compound as a white solid after purification by RP-HPLC (42-72% MeCN in 0.05% aqueous NH_4OH solution over 5 min) (19.2 mg, 80%). ^1H NMR (400 MHz, DMSO-d_6) δ 8.20 – 8.17 (m, 1H), 8.02 – 7.97 (m, 4H), 7.77 (d, *J* = 8.3 Hz, 2H), 7.70 (d, *J* = 7.1 Hz, 1H), 7.65 – 7.55 (m, 3H), 4.65 (s, 2H), 4.40 (s, 2H). HRMS (TOF, ES+), $C_{18}H_{17}F_5NS$ $[M+H]^+$ calc. mass 374.0996, found 374.1001.

3d (1-(4-(((Naphthalen-1-ylmethyl)amino)methyl)phenyl)pyrrolidin-2-one)

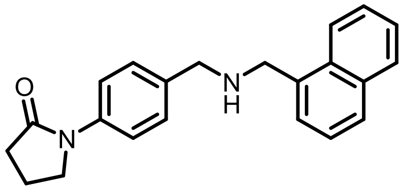

Followed General Procedure A with 1-naphthaldehyde (10 mg, 0.064 mmol, 1 equiv) and 1-(4-(aminomethyl)phenyl)pyrrolidin-2-one hydrochloride (22 mg, 0.096 mmol, 1.5 equiv) to give the title compound as a colorless oil after purification by RP-HPLC (10-40% MeCN in 0.1% aqueous TFA solution over 10 min) (2.5 mg, 12%). ^1H NMR (400 MHz, DMSO-d_6) δ 8.18 – 8.08 (m, 1H), 7.96 – 7.86 (m, 1H), 7.82 (d, *J* = 8.0 Hz, 1H), 7.65 – 7.58 (m, 2H), 7.55 – 7.43 (m, 4H), 7.41 – 7.37 (m, 2H), 4.13 (s, 2H), 3.89 – 3.78 (m, 4H), 2.50 – 2.44 (m, 2H), 2.05 (p, *J* = 7.7 Hz, 2H). HRMS (TOF, ES+), $C_{22}H_{23}N_2O$ $[M+H]^+$ calc. mass 331.1805, found 331.1805.

3e (*N*-(4-(3,5-Dimethyl-1*H*-pyrazol-1-yl)benzyl)-1-(naphthalen-1-yl)methanamine)

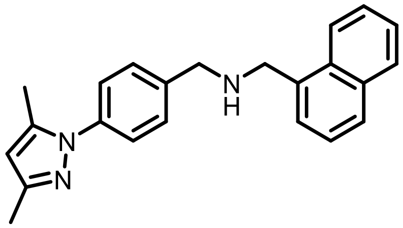

Followed General Procedure A with 1-naphthaldehyde (10 mg, 0.064 mmol, 1 equiv) and (4-(3,5-dimethyl-1*H*-pyrazol-1-yl)phenyl)methanamine (19 mg, 0.096 mmol, 1.5 equiv) to give the title compound as a colorless oil after purification by RP-HPLC (32-62% MeCN in 0.05% aqueous NH_4OH solution over 5 min) (12.5 mg, 57%). ^1H NMR (400 MHz, DMSO-d_6) δ 8.18 – 8.13 (m, 1H), 7.94 – 7.90 (m, 1H), 7.82 (d, *J* = 8.2 Hz, 1H), 7.56 – 7.42 (m, 8H), 6.05 (s, 1H), 4.16 (s, 2H), 3.86 (s, 2H), 2.27 (s, 3H), 2.17 (s, 3H). HRMS (TOF, ES+), $C_{23}H_{24}N_3$ $[M+H]^+$ calc. mass 342.1965, found 342.1968.

3f ((4-(((Naphthalen-1-ylmethyl)amino)methyl)phenyl)methanol)

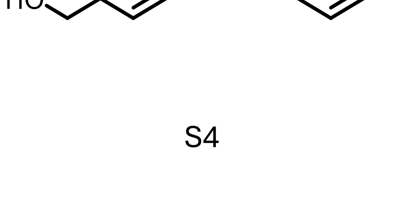

Followed General Procedure A with 1-naphthaldehyde (10 mg, 0.064 mmol, 1 equiv) and (4-(aminomethyl)phenyl)methanol (13 mg, 0.096 mmol, 1.5 equiv) to give the title compound as a colorless oil after purification by RP-HPLC (10-40% MeCN in 0.1% aqueous TFA solution over 10 min) (2.0 mg, 11%). ^{1}H NMR (400 MHz, DMSO-d_6) δ 8.14 – 8.11 (m, 1H), 7.94 – 7.90 (m, 1H), 7.83 (d, *J* = 8.1 Hz, 1H), 7.55 – 7.44 (m, 4H), 7.36 – 7.33 (m, 2H), 7.30 – 7.26 (m, 2H), 5.14 (t, *J* = 5.7 Hz, 1H), 4.48 (d, *J* = 5.6 Hz, 2H), 4.14 (s, 2H), 3.82 (s, 2H). HRMS (TOF, ES+), $C_{19}H_{20}NO$ [M+H]+ calc. mass 278.1539, found 278.1538.

3g (6-Methoxy-2-(naphthalen-1-ylmethyl)-1,2,3,4-tetrahydroisoquinoline)

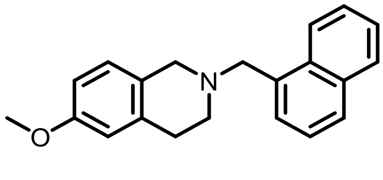

Followed General Procedure A with 1-naphthaldehyde (10 mg, 0.064 mmol, 1 equiv) and 6-methoxy-1,2,3,4-tetrahydroisoquinoline hydrochloride (19 mg, 0.096 mmol, 1.5 equiv) to give the title compound as a white solid after purification by RP-HPLC (50-80% MeCN in 0.05% aqueous NH4OH solution over 5 min) (13.1 mg, 67%). ^{1}H NMR (400 MHz, DMSO-d_6) δ 8.39 – 8.34 (m, 1H), 8.10 (d, *J* = 8.2 Hz, 1H), 8.05 (d, *J* = 7.9 Hz, 1H), 7.83 (d, *J* = 7.1 Hz, 1H), 7.70 – 7.60 (m, 3H), 7.09 (d, *J* = 8.7 Hz, 1H), 6.84 – 6.78 (m, 2H), 5.06 – 4.86 (m, 2H), 4.50 – 4.28 (m, 2H), 3.73 (s, 3H), 3.62 – 3.38 (m, 2H), 3.16 – 3.01 (s, 2H). HRMS (TOF, ES+), $C_{21}H_{22}NO$ [M+H]+ calc. mass 304.1696, found 304.1697.

3h (4-(Naphthalen-1-ylmethyl)-2,3,4,5-tetrahydrobenzo[*f*][1,4]oxazepane)

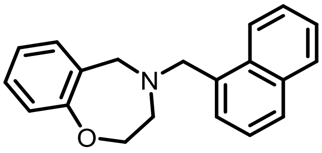

Followed General Procedure A with 1-naphthaldehyde (10 mg, 0.064 mmol, 1 equiv) and 2,3,4,5-tetrahydrobenzo[*f*][1,4]oxazepine hydrochloride (18 mg, 0.096 mmol, 1.5 equiv) to give the title compound as a colorless oil after purification by RP-HPLC (42-72% MeCN in 0.05% aqueous NH4OH solution over 5 min) (11.7 mg, 63%). ^{1}H NMR (400 MHz, DMSO-d_6) δ 8.27 – 8.21 (m, 1H), 7.93 – 7.89 (m, 1H), 7.85 (d, *J* = 8.1 Hz, 1H), 7.54 – 7.47 (m, 2H), 7.45 (dd, *J* = 8.1, 6.9 Hz, 1H), 7.38 (dd, *J* = 7.0, 1.4 Hz, 1H), 7.21 (td, *J* = 7.6, 1.8 Hz, 1H), 7.11 (dd, *J* = 7.4, 1.8 Hz, 1H), 7.03 – 6.98 (m, 2H), 4.06 – 4.03 (m, 2H), 4.01 (s, 2H), 3.85 (s, 2H), 3.01 – 2.99 (m, 2H). HRMS (TOF, ES+), $C_{20}H_{20}NO$ [M+H]+ calc. mass 290.1539, found 290.1541.

3i (*N*-(4-Bromobenzyl)-1-(naphthalen-1-yl)methanamine)

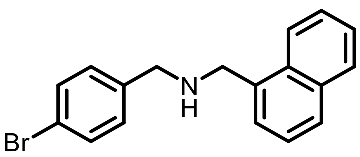

Followed General Procedure A with 1-naphthaldehyde (100 mg, 0.64 mmol, 1 equiv) and (4-bromophenyl)methanamine (179 mg, 0.96 mmol, 1.5 equiv) in DCM (5 mL) to give the title compound as a slightly yellow oil after purification by column chromatography (3-100% EtOAc in hexanes) (155 mg, 74%). ^{1}H NMR (400 MHz, DMSO-d_6) δ 8.16 – 8.10 (m, 1H), 7.94 – 7.89 (m, 1H), 7.81 (d, J = 8.3 Hz, 1H), 7.55 – 7.43 (m, 6H), 7.38 – 7.33 (m, 2H), 4.10 (s, 2H), 3.77 (s, 2H). HRMS (TOF, ES+), $C_{18}H_{17}BrN$ $[M+H]^+$ calc. mass 326.0539, found 326.0536.

General Procedure B: Cross-Coupling with Alcohols for the Synthesis of 3j and 7

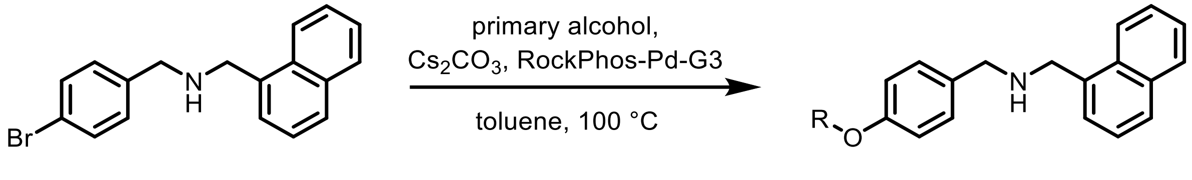

Intermediate **3i** (1 equiv), primary alcohol (5-10 equiv), cesium carbonate (2.5 equiv) and RockPhos-Pd-G3 (0.1 equiv, CAS: 2009020-38-4) were combined in a vial, which was sealed and placed under an atmosphere of N_2. Toluene (0.75 mL) was added, and the resulting reaction mixture was briefly stirred under vacuum and then stirred under an N_2 atmosphere at 100 °C overnight, after which time the reaction mixture was cooled to r.t., and solvents were concentrated. Solids were removed by syringe filtration, and crude residue was purified by RP-HPLC to give the title compounds.

3j (*N*-(4-(2-Methoxyethoxy)benzyl)-1-(naphthalen-1-yl)methanamine)

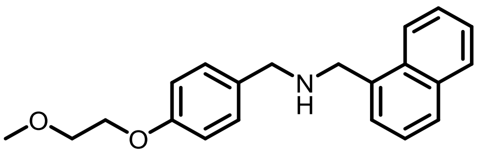

Followed General Procedure B with intermediate **3i** (14 mg, 0.043 mmol, 1 equiv) and 2-methoxyethanol (0.017 mL, 0.22 mmol, 5 equiv) to give the title compound as a colorless oil after purification by RP-HPLC (33-63% MeCN in 0.05% aqueous NH_4OH solution over 5 min) (4.2 mg, 30%). ^{1}H NMR (400 MHz, DMSO-d_6) δ 8.14 – 8.10 (m, 1H), 7.93 – 7.89 (m, 1H), 7.81 (d, J = 8.0 Hz, 1H), 7.54 – 7.43 (m, 4H), 7.31 – 7.26 (m, 2H), 6.92 – 6.87 (m, 2H), 4.10 (s, 2H), 4.08 – 4.05 (m, 2H), 3.74 (s, 2H), 3.66 – 3.63 (m, 2H), 3.30 (s, 3H). HRMS (TOF, ES+), $C_{21}H_{24}NO_2$ $[M+H]^+$ calc. mass 322.1802, found 322.1804.

7 (*N*-(4-(Methoxy-*d*$_3$)benzyl)-1-(naphthalen-1-yl)methanamine)

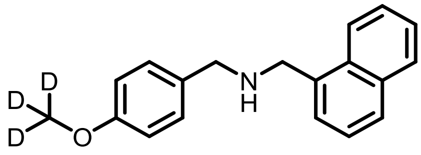

Followed General Procedure B with intermediate **3i** (20 mg, 0.61 mmol, 1 equiv) and methanol-d_4 (0.025 mL, 0.61 mmol, 10 equiv) to give the title compound as a colorless oil after purification by RP-HPLC (30-60% MeCN in 0.05% aqueous NH_4OH solution over 5 min) (7.2 mg, 42%). ^{1}H NMR (400 MHz, DMSO-d_6) δ 8.15 – 8.10 (m, 1H), 7.93 – 7.89 (m, 1H), 7.81 (d, J = 8.0

Hz, 1H), 7.54 – 7.43 (m, 4H), 7.31 – 7.28 (m, 2H), 6.90 – 6.87 (m, 2H), 4.09 (s, 2H), 3.73 (s, 2H). HRMS (TOF, ES+), $C_{19}H_{17}D_3NO$ $[M+H]^+$ calc. mass 281.1728, found 281.1730.

General Procedure C: Cross-Coupling with Isothiazolidine 1,1-dioxide for the Synthesis of 3k

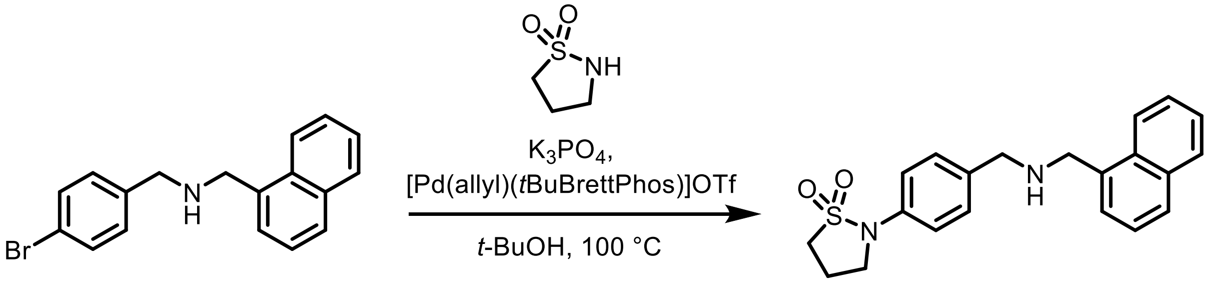

3k (2-(4-(((Naphthalen-1-ylmethyl)amino)methyl)phenyl)isothiazolidine 1,1-dioxide)

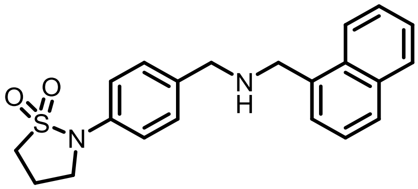

Intermediate **3i** (20 mg, 0.061 mmol, 1 equiv), isothiazolidine 1,1-dioxide (22 mg, 0.18 mmol, 3 equiv), potassium phosphate tribasic (22 mg, 0.10 mmol, 1.7 equiv) and [Pd(allyl)(*t*BuBrettPhos)]OTf (4.8 mg, 0.006 mmol, 0.1 equiv) were combined in a vial, which was sealed and placed under an atmosphere of N_2. *t*-BuOH (0.75 mL) was then added via syringe, and the resulting reaction mixture was stirred at 100 °C under an N_2 atmosphere overnight, after which time the reaction mixture was cooled to r.t. and solvents were concentrated. Solids were removed by syringe filtration, and crude residue was purified by RP-HPLC (22-52% MeCN in 0.05% aqueous NH_4OH solution over 5 min). Fractions containing product were extracted with DCM, and combined organic extracts were filtered through a hydrophobic phase separator and concentrated to give the title compound as a colorless oil (2.5 mg, 11%). ^{1}H NMR (400 MHz, DMSO-d_6) δ 8.15 – 8.11 (m, 1H), 7.94 – 7.89 (m, 1H), 7.83 (d, J = 8.1 Hz, 1H), 7.55 – 7.44 (m, 4H), 7.41 – 7.37 (m, 2H), 7.20 – 7.16 (m, 2H), 4.13 (s, 2H), 3.80 (s, 2H), 3.73 (t, J = 6.5 Hz, 2H), 3.49 (t, J = 7.4 Hz, 2H), 2.40 (p, J = 6.7 Hz, 2H). HRMS (TOF, ES+), $C_{21}H_{23}N_2O_2S$ $[M+H]^+$ calc. mass 367.1475, found 367.1480.

General Procedure D: Cross-Coupling with Dimethylphosphine Oxide for the Synthesis of 3l

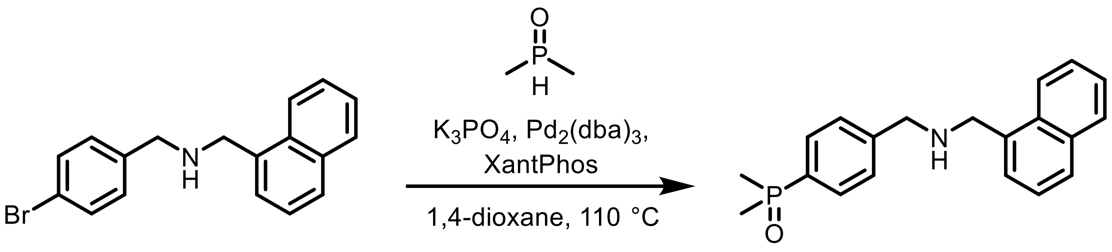

3l (Dimethyl(4-(((naphthalen-1-ylmethyl)amino)methyl)phenyl)phosphine oxide)

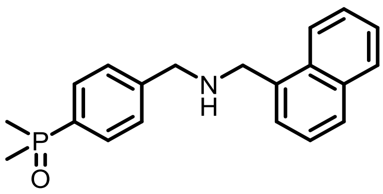

Intermediate **3i** (23 mg, 0.071 mmol, 1 equiv), dimethyphosphine oxide (17 mg, 0.21 mmol, 3 equiv), potassium phosphate tribasic (23 mg, 0.11 mmol, 1.5 equiv), XantPhos (4.1 mg, 0.007 mmol, 0.1 equiv) and $Pd_2(dba)_3$ (3.2 mg, 0.004 mmol, 0.05 equiv) were combined in a vial, which was sealed and placed under an atmosphere of N_2. 1,4-Dioxane (1 mL) was then added via syringe, and the resulting reaction mixture was stirred at 110 °C under an N_2 atmosphere overnight, after which time the reaction mixture was cooled to r.t. and solvents were concentrated. Solids were removed by syringe filtration, and crude residue was purified by RP-HPLC (7-37% MeCN in 0.1% aqueous TFA solution over 10 min). Fractions containing product were basified with sat. NaHCO3 solution and extracted with DCM, and combined organic extracts were filtered through a hydrophobic phase separator and concentrated to give the title compound as a colorless oil (8.1 mg, 36%). ^{1}H NMR (400 MHz, DMSO-d_6) δ 8.16 – 8.12 (m, 1H), 7.93 – 7.89 (m, 1H), 7.82 (d, J = 8.1 Hz, 1H), 7.72 (dd, J = 11.2, 7.9 Hz, 2H), 7.54 – 7.44 (m, 6H), 4.14 (s, 2H), 3.86 (s, 2H), 1.65 (s, 3H), 1.61 (s, 3H). HRMS (TOF, ES+), $C_{20}H_{23}NOP$ [M+H]+ calc. mass 324.1512, found 324.1515.

General Procedure E: Reductive Amination for the Synthesis of 5a-i

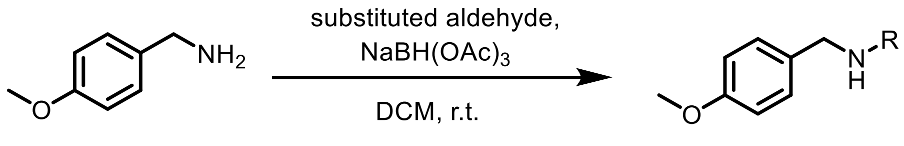

To a stirring solution of substituted aldehyde (1 equiv) and 4-methoxybenzylamine (1.5 equiv) in DCM (1 mL) was added sodium triacetoxyborohydride (2 equiv). The resulting reaction mixture was stirred at r.t. overnight or until completion, after which time sat. $NaHCO_3$ solution was slowly added. The aqueous layer was extracted with DCM, and combined organic extracts were

filtered through a hydrophobic phase separator and concentrated. Crude residue was purified by RP-HPLC to give the title compounds.

For acidic purifications (0.1% TFA), fractions containing product were basified with sat. $NaHCO_3$ solution and extracted with DCM. Combined organic extracts were filtered through a hydrophobic phase separator and concentrated to give the title compounds. For basic purifications (0.05% v/v NH_4OH), fractions containing product were extracted with DCM. Combined organic extracts were filtered through a hydrophobic phase separator and concentrated to give the title compounds. Alternatively, for basic purifications, fractions containing product were concentrated directly from the aqueous to give the title compounds.

5a (1-(Isoquinolin-1-yl)-*N*-(4-methoxybenzyl)methanamine)

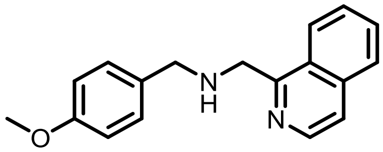

Followed General Procedure E with isoquinoline-1-carbaldehyde (10 mg, 0.064 mmol, 1 equiv) and 4-methoxybenzylamine (0.013 mL, 0.095 mmol, 1.5 equiv) to give the title compound as a colorless oil after purification by RP-HPLC (16-46% MeCN in 0.05% aqueous NH_4OH solution over 5 min) (5.9 mg, 33%). ^1H NMR (400 MHz, DMSO-d_6) δ 8.42 (d, *J* = 5.7 Hz, 1H), 8.29 (dd, *J* = 8.5, 1.1 Hz, 1H), 7.96 (d, *J* = 8.4 Hz, 1H), 7.78 – 7.63 (m, 3H), 7.30 – 7.27 (m, 2H), 6.90 – 6.86 (m, 2H), 4.27 (s, 2H), 3.75 (s, 2H), 3.73 (s, 3H). HRMS (TOF, ES+), $C_{18}H_{19}N_2O$ $[M+H]^+$ calc. mass 279.1492, found 279.1493.

5b (1-(Isoquinolin-4-yl)-*N*-(4-methoxybenzyl)methanamine)

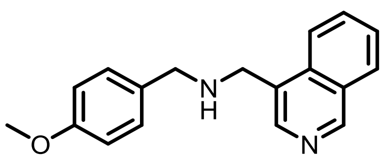

Followed General Procedure E with isoquinoline-4-carbaldehyde (10 mg, 0.064 mmol, 1 equiv) and 4-methoxybenzylamine (0.013 mL, 0.095 mmol, 1.5 equiv) to give the title compound as a colorless oil after purification by RP-HPLC (18-48% MeCN in 0.05% aqueous NH4OH solution over 5 min) (5.3 mg, 30%). ^1H NMR (400 MHz, DMSO-d_6) δ 9.22 (s, 1H), 8.44 (s, 1H), 8.17 (dd, *J* = 8.5, 1.1 Hz, 1H), 8.12 (dt, *J* = 8.1, 1.0 Hz, 1H), 7.79 (ddd, *J* = 8.4, 6.9, 1.4 Hz, 1H), 7.68 (ddd, *J* = 8.1, 6.9, 1.1 Hz, 1H), 7.30 – 7.27 (m, 2H), 6.91 – 6.87 (m, 2H), 4.07 (s, 2H), 3.73 (s, 3H), 3.73 (s, 2H). HRMS (TOF, ES+), $C_{18}H_{19}$N2O [M+H]+ calc. mass 279.1492, found 279.1493.

5c (1-(Isoquinolin-5-yl)-*N*-(4-methoxybenzyl)methanamine)

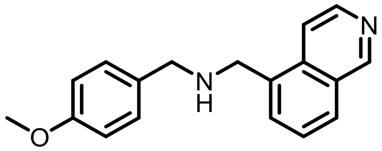

Followed General Procedure E with isoquinoline-5-carbaldehyde (10 mg, 0.064 mmol, 1 equiv) and 4-methoxybenzylamine (0.013 mL, 0.095 mmol, 1.5 equiv) to give the title compound as a colorless oil after purification by RP-HPLC (18-48% MeCN in 0.05% aqueous NH_4OH solution over 5 min) (5.9 mg, 33%). ^1H NMR (400 MHz, DMSO-d_6) δ 9.30 (s, 1H), 8.50 (d, J = 5.9 Hz, 1H), 8.01 (dt, J = 8.2, 1.1 Hz, 1H), 7.97 (dt, J = 5.9, 1.0 Hz, 1H), 7.77 (dd, J = 7.1, 1.2 Hz, 1H), 7.63 (dd, J = 8.2, 7.1 Hz, 1H), 7.31 – 7.27 (m, 2H), 6.91 – 6.87 (m, 2H), 4.10 (s, 2H), 3.73 (s, 3H), 3.72 (s, 2H). HRMS (TOF, ES+), $C_{18}H_{19}N_2O$ $[M+H]^+$ calc. mass 279.1492, found 279.1492.

5d (*N*-(4-Methoxybenzyl)-1-(quinolin-5-yl)methanamine)

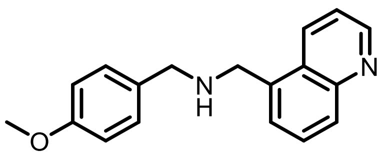

Followed General Procedure E with quinoline-5-carbaldehyde (10 mg, 0.064 mmol, 1 equiv) and 4-methoxybenzylamine (0.013 mL, 0.095 mmol, 1.5 equiv) to give the title compound as a colorless oil after purification by RP-HPLC (18-48% MeCN in 0.05% aqueous NH_4OH solution over 5 min) (5.7 mg, 32%). ^1H NMR (400 MHz, DMSO-d_6) δ 8.89 (dd, J = 4.1, 1.7 Hz, 1H), 8.58 (ddd, J = 8.5, 1.7, 0.9 Hz, 1H), 7.92 (dt, J = 8.5, 1.1 Hz, 1H), 7.69 (dd, J = 8.4, 7.0 Hz, 1H), 7.57 (dd, J = 7.0, 1.2 Hz, 1H), 7.53 (dd, J = 8.5, 4.1 Hz, 1H), 7.30 – 7.26 (m, 2H), 6.90 – 6.87 (m, 2H), 4.11 (s, 2H), 3.73 (s, 3H), 3.72 (s, 2H). HRMS (TOF, ES+), $C_{18}H_{19}N_2O$ $[M+H]^+$ calc. mass 279.1492, found 279.1494.

5e (*N*-(4-Methoxybenzyl)-1-(quinolin-4-yl)methanamine)

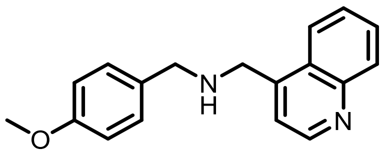

Followed General Procedure E with quinoline-4-carbaldehyde (10 mg, 0.064 mmol, 1 equiv) and 4-methoxybenzylamine (0.013 mL, 0.095 mmol, 1.5 equiv) to give the title compound as a colorless oil after purification by RP-HPLC (18-48% MeCN in 0.05% aqueous NH_4OH solution over 5 min) (5.1 mg, 29%). ^1H NMR (400 MHz, DMSO-d_6) δ 8.84 (d, J = 4.4 Hz, 1H), 8.13 (dd, J = 8.4, 1.7 Hz, 1H), 8.02 (dd, J = 8.5, 1.3 Hz, 1H), 7.74 (ddd, J = 8.4, 6.8, 1.4 Hz, 1H), 7.62 – 7.56 (m, 2H), 7.32 – 7.28 (m, 2H), 6.91 – 6.87 (m, 2H), 4.15 (s, 2H), 3.74 (s, 2H), 3.73 (s, 3H). HRMS (TOF, ES+), $C_{18}H_{19}N_2O$ $[M+H]^+$ calc. mass 279.1492, found 279.1492.

5f (1-(Imidazo[1,2-*a*]pyridin-8-yl)-*N*-(4-methoxybenzyl)methanamine)

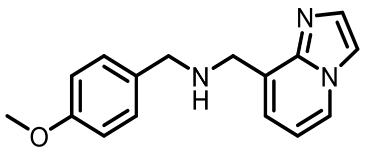

Followed General Procedure E with imidazo[1,2-*a*]pyridine-8-carbaldehyde (10 mg, 0.068 mmol, 1 equiv) and 4-methoxybenzylamine (0.013 mL, 0.10 mmol, 1.5 equiv) to give the title compound as a colorless oil after purification by RP-HPLC (12-42% MeCN in 0.05% aqueous

NH_4OH solution over 5 min) (5.2 mg, 28%). ^1H NMR (400 MHz, DMSO-d_6) δ 8.44 (dd, *J* = 6.8, 1.2 Hz, 1H), 7.93 (d, *J* = 1.2 Hz, 1H), 7.52 (d, *J* = 1.2 Hz, 1H), 7.28 – 7.24 (m, 2H), 7.23 – 7.19 (m, 1H), 6.89 – 6.84 (m, 3H), 3.98 (s, 2H), 3.73 (s, 3H), 3.69 (s, 2H). HRMS (TOF, ES+), $C_{16}H_{18}N_3O$ $[M+H]^+$ calc. mass 268.1444, found 268.1443.

5g (1-(2,2-Difluorobenzo[*d*][1,3]dioxol-4-yl)-*N*-(4-methoxybenzyl)methanamine)

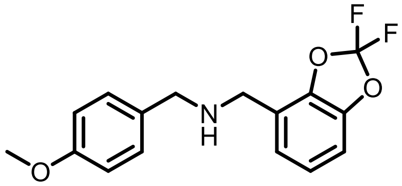

Followed General Procedure E with 2,2-difluorobenzo[*d*][1,3]dioxole-4-carbaldehyde (10 mg, 0.054 mmol, 1 equiv) and 4-methoxybenzylamine (0.011 mL, 0.081 mmol, 1.5 equiv) to give the title compound as a colorless oil after purification by RP-HPLC (32-62% MeCN in 0.05% aqueous NH_4OH solution over 5 min) (7.1 mg, 43%). ^1H NMR (400 MHz, DMSO-d_6) δ 7.28 – 7.22 (m, 4H), 7.19 – 7.15 (m, 1H), 6.89 – 6.85 (m, 2H), 3.73 (s, 3H), 3.72 (s, 2H), 3.62 (s, 2H). HRMS (TOF, ES+), $C_{16}H_{16}F_2NO_3$ $[M+H]^+$ calc. mass 308.1093, found 308.1095.

5h (1-(2,2-Difluorobenzo[*d*][1,3]dioxol-5-yl)-*N*-(4-methoxybenzyl)methanamine)

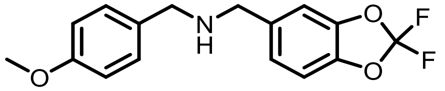

Followed General Procedure E with 2,2-difluorobenzo[*d*][1,3]dioxole-5-carbaldehyde (15 mg, 0.081 mmol, 1 equiv) and 4-methoxybenzylamine (0.016 mL, 0.12 mmol, 1.5 equiv) to give the title compound as a colorless oil after purification by RP-HPLC (28-58% MeCN in 0.05% aqueous NH_4OH solution over 5 min) (9.4 mg, 38%). ^1H NMR (400 MHz, DMSO-d_6) δ 7.37 (d, *J* = 1.6 Hz, 1H), 7.31 (d, *J* = 8.2 Hz, 1H), 7.25 – 7.22 (m, 2H), 7.14 (dd, *J* = 8.3, 1.6 Hz, 1H), 6.88 – 6.84 (m, 2H), 3.72 (s, 3H), 3.66 (s, 2H), 3.58 (s, 2H). HRMS (TOF, ES+), $C_{16}H_{16}F_2NO_3$ $[M+H]^+$ calc. mass 308.1093, found 308.1094.

5i (*N*-(4-Bromobenzyl)-1-(4-methoxyphenyl)methanamine)

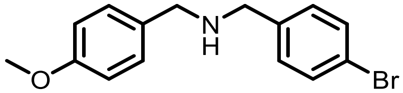

Followed General Procedure E with 4-bromobenzaldehyde (1.50 g, 8.11 mmol, 1 equiv) and 4-methoxybenzylamine (1.59 mL, 12.2 mmol, 1.5 equiv) in DCM (40 mL) to give the title compound as a yellow oil after purification by column chromatography (0-100% EtOAc in hexanes) (1.73 g, 70%). ^1H NMR (400 MHz, DMSO-d_6) δ 7.51 – 7.47 (m, 2H), 7.31 – 7.28 (m, 2H), 7.25 – 7.21 (m, 2H), 6.88 – 6.85 (m, 2H), 3.72 (s, 3H), 3.61 (s, 2H), 3.58 (s, 2H). HRMS (TOF, ES+), $C_{15}H_{17}BrNO$ $[M+H]^+$ calc. mass 306.0488, found 306.0486.

General Procedure F: Alkylation for the Synthesis of 5j and 5k

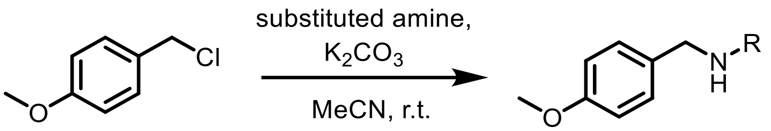

To a stirring suspension of substituted amine (1.2 equiv) and potassium carbonate (2 equiv) in MeCN (1 mL) was added 4-methoxybenzyl chloride (1 equiv). The resulting reaction mixture was stirred at r.t. overnight or until completion, after which time solvents were concentrated. Crude residue was taken up in DCM and H_2O, and the aqueous layer was extracted with DCM. Combined organic extracts were filtered through a hydrophobic phase separator and concentrated. Crude residue was purified by RP-HPLC to give the title compounds.

5j (1-([1,1'-Biphenyl]-3-yl)-*N*-(4-methoxybenzyl)methanamine)

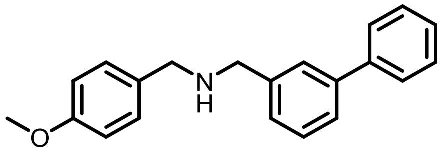

Followed General Procedure F with 4-methoxybenzyl chloride (0.013 mL, 0.096 mmol, 1 equiv) and [1,1'-biphenyl]-3-ylmethanamine (21 mg, 0.11 mmol, 1.2 equiv) to give the title compound as a colorless oil after purification by RP-HPLC (38-68% MeCN in 0.05% aqueous NH4OH solution over 5 min) (2.1 mg, 7%). ^{1}H NMR (400 MHz, DMSO-d_6) δ 7.70 – 7.65 (m, 3H), 7.58 (d, *J* = 7.6 Hz, 1H), 7.49 – 7.43 (m, 3H), 7.40 – 7.35 (m, 2H), 7.34 – 7.30 (m, 2H), 6.94 – 6.90 (m, 2H), 3.89 (s, 2H), 3.81 (s, 2H), 3.74 (s, 3H). HRMS (TOF, ES+), $C_{21}H_{22}NO$ [M+H]+ calc. mass 304.1696, found 304.1694.

5k (1-([1,1'-Biphenyl]-4-yl)-*N*-(4-methoxybenzyl)methanamine)

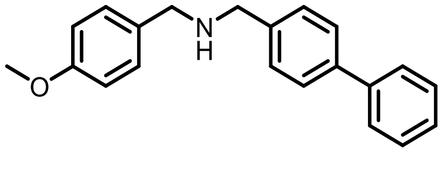

Followed General Procedure F with 4-methoxybenzyl chloride (0.013 mL, 0.096 mmol, 1 equiv) and [1,1'-biphenyl]-4-ylmethanamine (21 mg, 0.11 mmol, 1.2 equiv) to give the title compound as a colorless oil after purification by RP-HPLC (37-67% MeCN in 0.05% aqueous NH4OH solution over 5 min) (7.3 mg, 25%). ^{1}H NMR (400 MHz, DMSO-d_6) δ 7.67 – 7.61 (m, 4H), 7.4 8 – 7.43 (m, 4H), 7.37 – 7.33 (m, 1H), 7.30 – 7.27 (m, 2H), 6.91 – 6.87 (m, 2H), 3.74 (s, 2H), 3.74 (s, 3H), 3.68 (s, 2H). HRMS (TOF, ES+), $C_{21}H_{22}NO$ [M+H]+ calc. mass 304.1696, found 304.1701.

General Procedure G: Suzuki-Miyaura Coupling for the Synthesis of 5m-u

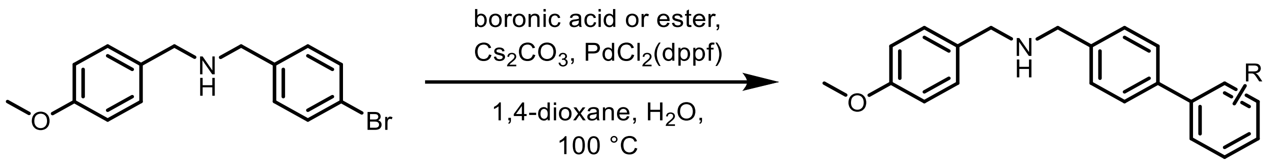

Intermediate **5i** (1 equiv), boronic acid or ester (1.2 equiv), cesium carbonate (2 equiv) and $PdCl_2$(dppf) (0.1 eq) were combined in 1,4-dioxane (0.75 mL) and water (0.075 mL). The resulting reaction mixture was sparged with N_2 and then heated to 100 °C and stirred for 3 h, or until completion. The reaction mixture was cooled to r.t. and diluted with EtOAc. Solids were removed by filtration, and filtrate was concentrated. Crude residue was purified by RP-HPLC to give the title compounds.

5m (*N*-(4-Methoxybenzyl)-1-(4-(pyridin-3-yl)phenyl)methanamine)

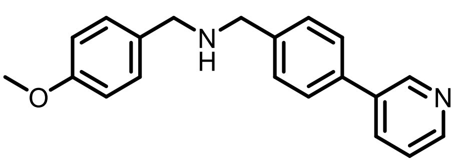

Followed General Procedure G with intermediate **5i** (20 mg, 0.07 mmol, 1 equiv) and 3-(4,4,5,5-tetramethyl-1,3,2-dioxaborolan-2-yl)pyridine (16.1 mg, 0.08 mmol, 1.2 equiv) to give the title compound as a tan oil after purification by RP-HPLC (5-50% MeCN in 0.1% aqueous TFA solution over 10 min) (8.2 mg, 41%). ^{1}H NMR (400 MHz, DMSO-d_6) δ 8.89 (dd, *J* = 2.4, 0.9 Hz, 1H), 8.55 (dd, *J* = 4.8, 1.6 Hz, 1H), 8.06 (ddd, *J* = 7.9, 2.5, 1.6 Hz, 1H), 7.69 – 7.66 (m, 2H), 7.49 – 7.45 (m, 3H), 7.28 – 7.25 (m, 2H), 6.90 – 6.86 (m, 2H), 3.73 (s, 3H), 3.71 (s, 2H), 3.63 (s, 2H). HRMS (TOF, ES+) calculated for $C_{20}H_{21}N_2O$ $[M+H]^+$ = 305.1648, found = 305.1654.

5n (*N*-(4-Methoxybenzyl)-1-(4-(pyridin-4-yl)phenyl)methanamine)

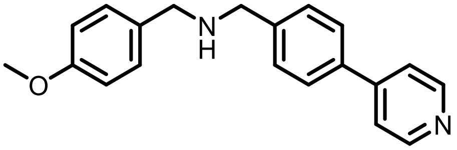

Followed General Procedure G with intermediate **5i** (20 mg, 0.07 mmol, 1 equiv) and 4-(4,4,5,5-tetramethyl-1,3,2-dioxaborolan-2-yl)pyridine (16.1 mg, 0.08 mmol, 1.2 equiv) to give the title compound as a tan oil after purification by RP-HPLC (5-50% MeCN in 0.1% aqueous TFA solution over 10 min) (11 mg, 55%).^{1}H NMR (400 MHz, DMSO-d_6) δ 8.63 – 8.61 (m, 2H), 7.78 – 7.74 (m, 2H), 7.71 – 7.69 (m, 2H), 7.50 – 7.46 (m, 2H), 7.28 – 7.24 (m, 2H), 6.90 – 6.86 (m, 2H), 3.73 (s, 3H), 3.72 (s, 2H), 3.62 (s, 2H). HRMS (TOF, ES+) $C_{20}H_{21}N_2O$ $[M+H]^+$ calc. mass 305.1648, found 305.1652.

5o (1-(2'-Fluoro-[1,1'-biphenyl]-4-yl)-*N*-(4-methoxybenzyl)methanamine)

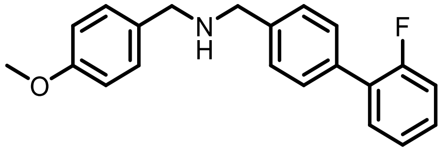

Followed General Procedure G with intermediate **5i** (20 mg, 0.07 mmol, 1 equiv) and 2-fluorophenylboronic acid (11 mg, 0.08 mmol, 1.2 equiv) to give the title compound as a tan oil after purification by RP-HPLC (25-65% MeCN in 0.1% aqueous TFA solution over 10 min) (9.4 mg, 45%). ^{1}H NMR (400 MHz, DMSO-d_6) δ 7.54 – 7.47 (m, 3H), 7.46 – 7.38 (m, 3H), 7.33 – 7.25 (m, 4H), 6.90 – 6.86 (m, 2H), 3.73 (s, 3H), 3.71 (s, 2H), 3.64 (s, 2H). HRMS (TOF, ES+) $C_{21}H_{21}FNO$ $[M+H]^+$ calc. mass 322.1602, found 322.1603.

5p (1-(3'-Fluoro-[1,1'-biphenyl]-4-yl)-*N*-(4-methoxybenzyl)methanamine)

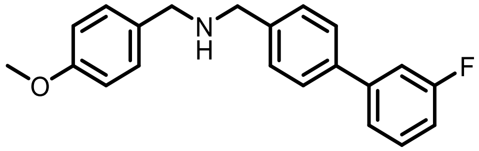

Followed General Procedure G with intermediate **5i** (20 mg, 0.07 mmol, 1 equiv) and 3-fluorophenylboronic acid (11 mg, 0.08 mmol, 1.2 equiv) to give the title compound as a clear oil after purification by RP-HPLC (25-65% MeCN in 0.1% aqueous TFA solution over 10 min) (11.7 mg, 56%). ^{1}H NMR (400 MHz, DMSO-d_6) δ 7.67 – 7.64 (m, 2H), 7.53 – 7.46 (m, 3H), 7.45 – 7.41 (m, 2H), 7.28 – 7.24 (m, 2H), 7.20 – 7.15 (m, 1H), 6.90 – 6.86 (m, 2H), 3.73 (s, 3H), 3.70 (s, 2H), 3.62 (s, 2H). HRMS (TOF, ES+) $C_{21}H_{21}FNO$ $[M+H]^+$ calc. mass 322.1602, found 322.1609.

5q (1-(4'-Fluoro-[1,1'-biphenyl]-4-yl)-*N*-(4-methoxybenzyl)methanamine)

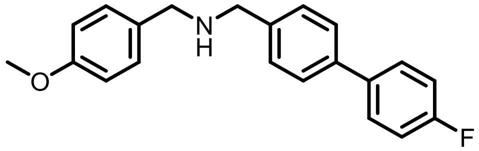

Followed General Procedure G with intermediate **5i** (20 mg, 0.07 mmol, 1 equiv) and 4-fluorophenylboronic acid (11 mg, 0.08 mmol, 1.2 equiv) to give the title compound as a yellow oil after purification by RP-HPLC (25-65% MeCN in 0.1% aqueous TFA solution over 10 min) (12 mg, 57%). ^{1}H NMR (400 MHz, DMSO-d_6) δ 7.72– 7.66 (m, 2H), 7.60 – 7.57 (m, 2H), 7.43 – 7.39 (m, 2H), 7.30 – 7.24 (m, 4H), 6.90 – 6.86 (m, 2H), 3.73 (s, 3H), 3.69 (s, 2H), 3.63 (s, 2H). HRMS (TOF, ES+) $C_{21}H_{21}FNO$ $[M+H]^+$ calc. mass 322.1602, found 322.1607.

5r (*N*-(4-Methoxybenzyl)-1-(3'-methyl-[1,1'-biphenyl]-4-yl)methanamine)

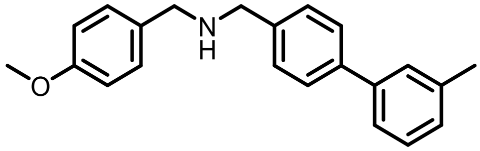

Followed General Procedure G with intermediate **5i** (20 mg, 0.07 mmol, 1 equiv) and m-tolylboronic acid (10.7 mg, 0.08 mmol, 1.2 equiv) to give the title compound as a brown oil after purification by RP-HPLC (25-65% MeCN in 0.1% aqueous TFA solution over 10 min) (9.4 mg, 45%). ^{1}H NMR (400 MHz, DMSO-d_6) δ 7.61 – 7.58 (m, 2H), 7.47 – 7.39 (m, 4H), 7.33 (t, *J* = 7.6 Hz, 1H), 7.29 – 7.25 (m, 2H), 7.18 – 7.14 (m, 1H), 6.90 – 6.87 (m, 2H), 3.73 (s, 3H), 3.70 (s, 2H), 3.64 (s, 2H), 2.37 (s, 3H). HRMS (TOF, ES+) $C_{22}H_{24}NO$ $[M+H]^+$ calc. mass 318.1852, found 318.1857.

5s (1-(3'-Methoxy-[1,1'-biphenyl]-4-yl)-*N*-(4-methoxybenzyl)methanamine)

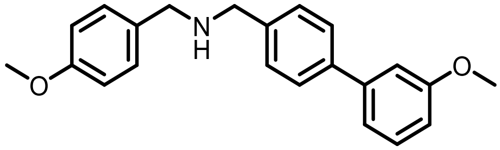

Followed General Procedure G with intermediate **5i** (15 mg, 0.05 mmol, 1 equiv) and 3-methoxyphenylboronic acid (8.9 mg, 0.06 mmol, 1.2 equiv) to give the title compound as a yellow oil after purification by RP-HPLC (25-65% MeCN in 0.1% aqueous TFA solution over 10 min) (10.8 mg, 66%).^{1}H NMR (400 MHz, DMSO-d_6) δ 7.63 – 7.59 (m, 2H), 7.43 – 7.40 (m, 2H), 7.36 (t, *J* = 7.9 Hz, 1H), 7.28 – 7.25 (m, 2H), 7.22 (ddd, *J* = 7.7, 1.7, 1.0 Hz, 1H), 7.17 (dd, *J* = 2.6, 1.6 Hz, 1H), 6.92 (ddd, *J* = 8.2, 2.6, 0.9 Hz, 1H), 6.90 – 6.86 (m, 2H), 3.82 (s, 3H), 3.73 (s, 3H), 3.70 (s, 2H), 3.63 (s, 2H). HRMS (TOF, ES+) $C_{22}H_{24}NO_2$ $[M+H]^+$ calc. mass 334.1802, found 334.1805.

5t (4'-(((4-Methoxybenzyl)amino)methyl)-[1,1'-biphenyl]-3-carbonitrile)

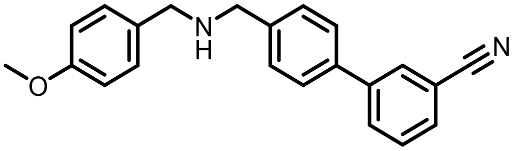

Followed General Procedure G with intermediate **5i** (20 mg, 0.07 mmol, 1 equiv) and (3-cyanophenyl)boronic acid (8.6 mg, 0.06 mmol, 1.2 equiv) to give the title compound as a yellow oil after purification by RP-HPLC (20-60% MeCN in 0.1% aqueous TFA solution over 10 min) (9.4 mg, 58%). ^{1}H NMR (400 MHz, DMSO-d_6) δ 8.15 (t, *J* = 1.7 Hz, 1H), 8.02 (ddd, *J* = 8.0, 1.9, 1.1 Hz, 1H), 7.81 (dt, *J* = 7.7, 1.3 Hz, 1H), 7.72 – 7.64 (m, 3H), 7.48 – 7.44 (m, 2H), 7.28 – 7.25 (m, 2H), 6.90 – 6.86 (m, 2H), 3.73 (s, 3H), 3.71 (s, 2H), 3.63 (s, 2H). HRMS (TOF, ES+) $C_{22}H_{21}N_2O$ $[M+H]^+$ calc. mass 329.1648, found 329.1654.

5u (*N*-(4-Methoxybenzyl)-1-(4-(1-methyl-1*H*-pyrazol-4-yl)phenyl)methanamine)

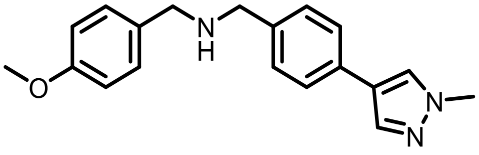

Followed General Procedure G with intermediate **5i** (20 mg, 0.06 mmol, 1 equiv) and 1-methyl-4-(4,4,5,5-tetramethyl-1,3,2-dioxaborolan-2-yl)-1H-pyrazole (16.3 mg, 0.08 mmol, 1.2 equiv) to give the title compound as an orange oil after purification by RP-HPLC (25-65% MeCN

in 0.05% aqueous NH_4OH solution over 10 min) (9.8 mg, 49%). ^{1}H NMR (400 MHz, DMSO) δ 8.09 (s, 1H), 7.82 (s, 1H), 7.51 – 7.47 (m, 2H), 7.31 – 7.28 (m, 2H), 7.27 – 7.23 (m, 2H), 6.89 – 6.86 (m, 2H), 3.85 (s, 3H), 3.73 (s, 3H), 3.63 (s, 2H), 3.61 (s, 2H). HRMS (TOF, ES+) $C_{19}H_{22}N_3O$ $[M+H]^+$ calc. mass 308.1757, found 308.1762.

General Procedure H: Buchwald-Hartwig Coupling for the Synthesis of 5v-y

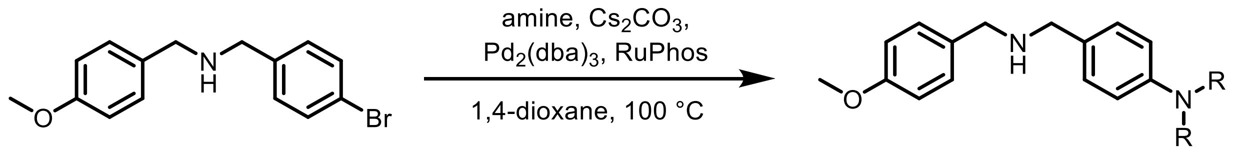

Intermediate **5i** (1 equiv), amine (1.2 eq), cesium carbonate (2 equiv), $Pd_2(dba)_3$ (0.1 equiv) and RuPhos (0.1 equiv) were combined in a vial, which was sealed and placed under an atmosphere of N_2. 1,4-Dioxane (0.5 mL) was then added via syringe, and the resulting reaction mixture was stirred under an atmosphere of N_2 at 100 °C for 3 h or until completion, after which time the reaction mixture was cooled to r.t. The reaction mixture was diluted with EtOAc and passed through a plug of Celite. Solvents were concentrated, and crude residue was purified by RP-HPLC to give the title compounds.

5v (*N*-(4-Methoxybenzyl)-1-(4-(pyrrolidin-1-yl)phenyl)methanamine)

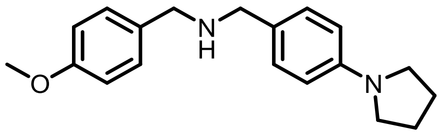

Followed General Procedure H with **5i** (20 mg, 0.065 mmol, 1 equiv) and pyrrolidine (0.007 mL, 0.078 mmol, 1.2 equiv) to give the title compound as a dark oil after purification by RP-HPLC (16-51% MeCN in 0.1% aqueous TFA solution over 5 min) (16 mg, 83%). ^{1}H NMR (400 MHz, DMSO-d_6) δ 7.25 – 7.22 (m, 2H), 7.13 – 7.09 (m, 2H), 6.89 – 6.85 (m, 2H), 6.50 – 6.46 (m, 2H), 3.73 (s, 3H), 3.59 (s, 2H), 3.55 (s, 2H), 3.20 – 3.17 (m, 4H), 1.95 – 1.92 (m, 4H). HRMS (TOF, ES+), $C_{19}H_{23}N_2O$ $[M-H]^+$ calc. mass 295.1805, found 295.1796.

5w (*N*-(4-Methoxybenzyl)-1-(4-(piperidin-1-yl)phenyl)methanamine)

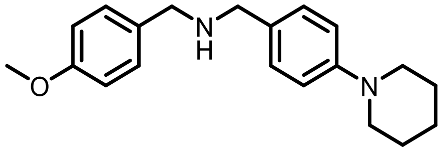

Followed General Procedure H with **5i** (20 mg, 0.065 mmol, 1 equiv) and piperidine (0.008 mL, 0.078 mmol, 1.2 equiv) to give the title compound as a dark oil after purification by RP-HPLC (4-39% MeCN in 0.1% aqueous TFA solution over 5 min) (15 mg, 74%). ^{1}H NMR (400 MHz, DMSO-d_6) δ 7.25 – 7.21 (m, 2H), 7.16 – 7.12 (m, 2H), 6.88 – 6.83 (m, 4H), 3.72 (s, 3H), 3.57 (s, 2H), 3.53 (s, 2H), 3.09 – 3.06 (m, 4H), 1.63 – 1.57 (m, 4H), 1.55 – 1.48 (m, 2H). HRMS (TOF, ES+), $C_{20}H_{25}N_2O$ $[M-H]^+$ calc. mass 311.2118, found 311.2112.

5x (*N*-(4-(Azepan-1-yl)benzyl)-1-(4-methoxyphenyl)methanamine)

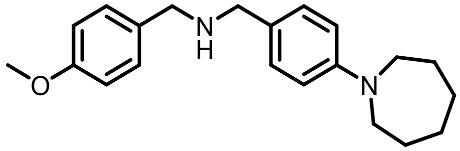

Followed General Procedure H with **5i** (20 mg, 0.065 mmol, 1 equiv) and azepane (0.009 mL, 0.078 mmol, 1.2 equiv) to give the title compound as a dark oil after purification by RP-HPLC (21-56% MeCN in 0.1% aqueous TFA solution over 5 min) (14 mg, 66%). ^{1}H NMR (400 MHz, DMSO-d_6) δ 7.27 – 7.23 (m, 2H), 7.11 – 7.07 (m, 2H), 6.89 – 6.85 (m, 2H), 6.62 – 6.59 (m, 2H), 3.73 (s, 3H), 3.63 (s, 2H), 3.54 (s, 2H), 3.42 (t, *J* = 6.0 Hz, 4H), 1.75 – 1.64 (m, 4H), 1.48 – 1.40 (m, 4H). HRMS (TOF, ES+), $C_{21}H_{29}N_2O$ $[M+H]^+$ calc. mass 325.2274, found 325.2266.

5y (*N*-(4-Methoxybenzyl)-1-(4-morpholinophenyl)methanamine)

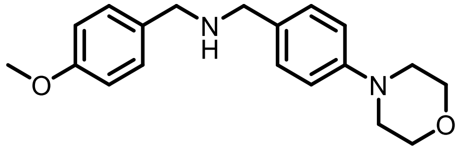

Followed General Procedure H with **5i** (20 mg, 0.065 mmol, 1 equiv) and morpholine (0.007 mL, 0.078 mmol, 1.2 equiv) to give the title compound as a dark oil after purification RP-HPLC (9-44% MeCN in 0.1% aqueous TFA solution over 5 min) 15 mg, 74%). ^{1}H NMR (400 MHz, DMSO-d_6) δ 7.26 – 7.22 (m, 2H), 7.20 – 7.17 (m, 2H), 6.90 – 6.85 (m, 4H), 3.74 – 3.71 (m, 7H), 3.59 (d, *J* = 9.7 Hz, 4H), 3.07 – 3.05 (m, 4H). HRMS (TOF, ES+), $C_{19}H_{25}N_2O_2$ $[M+H]^+$ calc. mass 313.1911, found 313.1911.

General Procedure I: Cross-Coupling with 1,2-Thiazinane 1,1-dioxide for the Synthesis of 5z

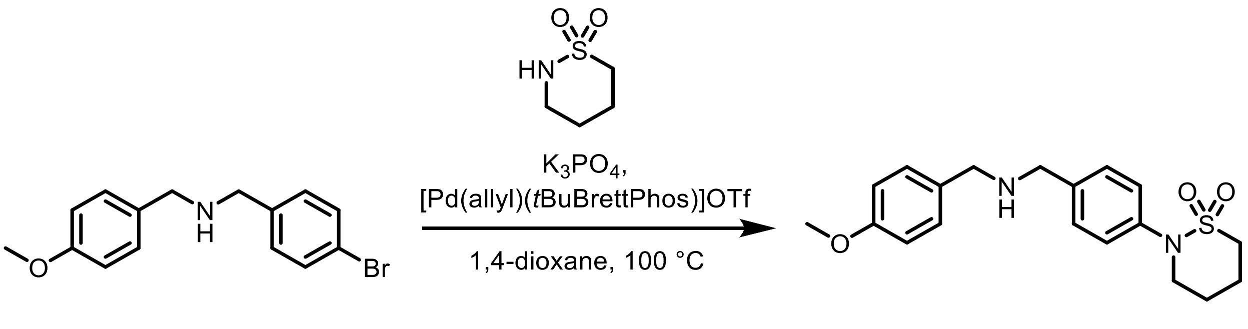

5z (2-(4-(((4-Methoxybenzyl)amino)methyl)phenyl)-1,2-thiazinane 1,1-dioxide)

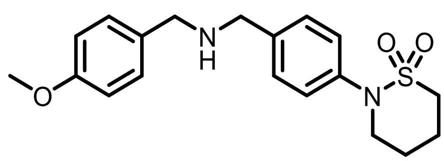

Intermediate **5i** (20 mg, 0.065 mmol, 1 equiv), 1,2-thiazinane 1,1-dioxide (26 mg, 0.20 mmol, 3 equiv), potassium phosphate tribasic (24 mg, 0.11 mmol, 1.7 equiv) and [Pd(allyl)(*t*BuBrettPhos)]OTf (5.1 mg, 0.007 mmol, 0.1 equiv) were combined in a vial, which was sealed and placed under an atmosphere of N_2. 1,4-Dioxane (0.75 mL) was then added via syringe, and the resulting reaction mixture was stirred at 100 °C under an N_2 atmosphere overnight, after which time the reaction mixture was cooled to r.t., diluted with EtOAc, and filtered through a pad of Celite. Solvents were concentrated under vacuum and crude residue was purified by RP-HPLC (11-46% MeCN in 0.1% aqueous TFA solution over 5 min) to give the title compound as a yellow oil (10 mg, 42%). ^{1}H NMR (400 MHz, DMSO-d_6) δ 7.36 – 7.31 (m, 2H), 7.28 – 7.22 (m, 4H), 6.89 – 6.84 (m, 2H), 3.73 (s, 3H), 3.65 – 3.59 (m, 6H), 3.28 – 3.25 (m, 2H), 2.18 – 2.12 (m, 2H), 1.82 – 1.77 (m, 2H). HRMS (TOF, ES+), $C_{19}H_{25}N_2O_3S$ $[M+H]^+$ calc. mass 361.1580, found 361.1582.

General Procedure J: Borylation/Suzuki-Miyaura Coupling for the Synthesis of 5l

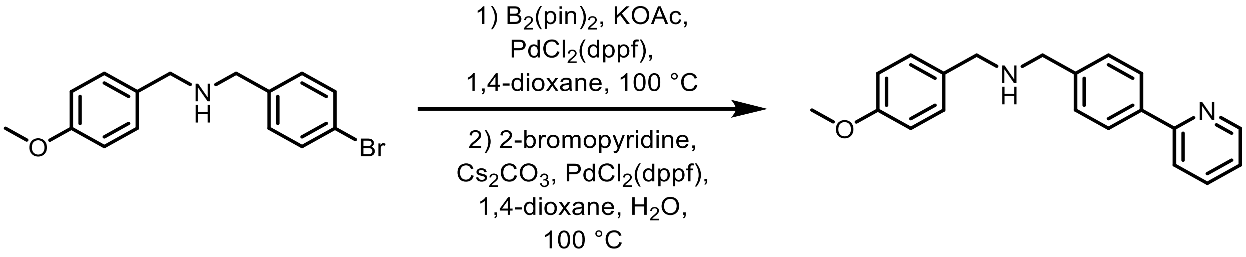

5l (*N*-(4-Methoxybenzyl)-1-(4-(pyridin-2-yl)phenyl)methanamine)

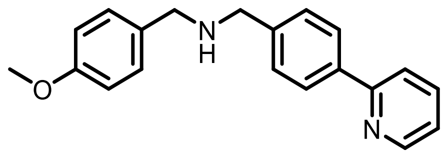

Intermediate **5i** (35 mg, 0.11 mmol, 1 equiv), bis(pinacolato)diboron (44 mg, 0.17 mmol, 1.5 equiv), potassium acetate (34 mg, 0.34 mmol, 3 equiv) and $PdCl_2$(dppf).DCM (9.4 mg, 0.011 mmol, 0.1 equiv) were combined in 1,4-dioxane (1 mL). The reaction mixture was sparged with N_2 and then heated to 100 °C for 2 h. The resulting reaction mixture was cooled to r.t., diluted with EtOAc, and filtered through a pad of Celite. The filtrate was concentrated to afford the intermediate pinacol boronate, which was carried forward directly without further purification (N-

(4-methoxybenzyl)-1-(4-(4,4,5,5-tetramethyl-1,3,2-dioxaborolan-2-yl)phenyl)methanamine). ES-MS [M+H]+ = 354.

The pinacol boronate intermediate (1 equiv) was combined with 2-bromopyridine (11 mg, 0.068 mmol, 1.2 equiv), cesium carbonate (37 mg, 0.11 mmol, 2 equiv) and $PdCl_2$(dppf).DCM (4.6 mg, 0.006 mmol, 0.1 equiv). 1,4-Dioxane (0.75 mL) and H_2O (0.075 mL) were then added, and the reaction mixture was sparged with N_2, and stirred at 100 °C under an N_2 atmosphere overnight. The resulting reaction mixture was cooled to r.t. and diluted with EtOAc. Solids were removed by filtration, and filtrate was concentrated. Crude residue was purified by RP-HPLC (5-50% MeCN in 0.1% aqueous TFA solution over 10 min). Fractions containing product were basified with sat. $NaHCO_3$ solution and extracted with EtOAc. Combined organic extracts were passed through a hydrophobic phase separator and concentrated to give the title compound as a clear oil (2.8 mg, 16%). ^{1}H NMR (400 MHz, DMSO-d_6) δ 8.65 (ddd, *J* = 4.8, 1.9, 0.9 Hz, 1H), 8.06 – 8.02 (m, 2H), 7.95 (dt, *J* = 8.0, 1.1 Hz, 1H), 7.87 (td, *J* = 7.7, 1.9 Hz, 1H), 7.48 – 7.44 (m, 2H), 7.33 (ddd, *J* = 7.4, 4.8, 1.1 Hz, 1H), 7.30 – 7.26 (m, 2H), 6.91 – 6.87 (m, 2H), 3.75 (s, 2H), 3.74 (s, 3H), 3.67 (s, 2H). HRMS (TOF, ES+) $C_{20}H_{21}N_2O$ [M+H]+ calc. mass 305.1648, found 305.1652.

Characterization of Deuterated Analogs of 5s (9, 11, 13)

8 *N*-(4-Bromobenzyl)-1-(3'-methoxy-[1,1'-biphenyl]-4-yl)methanamine

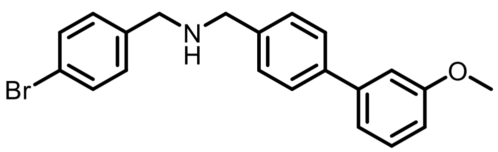

Followed General Procedure E with 3'-methoxy-[1,1'-biphenyl]-4-carbaldehyde (50 mg, 0.24 mmol, 1 equiv) and (4-bromophenyl)methanamine (65.7 mg, 0.35 mmol, 1.5 equiv) to give the title compound as a tan gel after purification by column chromatography (0-50% EtOAc in hexanes) (58.2 mg, 65%). ^{1}H NMR (400 MHz, DMSO) δ 7.64 – 7.59 (m, 2H), 7.53 – 7.49 (m, 2H), 7.44 – 7.40 (m, 2H), 7.39 – 7.31 (m, 3H), 7.22 (ddd, *J* = 7.7, 1.7, 1.0 Hz, 1H), 7.17 (dd, *J* = 2.6, 1.7 Hz, 1H), 6.92 (ddd, *J* = 8.2, 2.6, 0.9 Hz, 1H), 3.82 (s, 3H), 3.71 (s, 2H), 3.68 (s, 2H). HRMS (TOF, ES+), $C_{21}H_{21}BrNO$ [M+H]+ calc. mass 382.0801, found 382.0804.

9 *N*-(4-(Methoxy-d_3)benzyl)-1-(3'-methoxy-[1,1'-biphenyl]-4-yl)methanamine

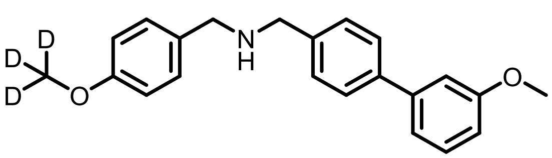

Followed General Procedure B with intermediate **8** (25 mg, 0.065 mmol, 1 equiv) and methanol-d_4 (0.027 mL, 0.65 mmol, 10 equiv) to give the title compound as a tan oil after purification by column chromatography (0-60% EtOAc in hexanes) (15.6 mg, 71%). ^{1}H NMR (400 MHz, DMSO) δ 7.64 – 7.58 (m, 2H), 7.44 – 7.39 (m, 2H), 7.36 (t, *J* = 7.9 Hz, 1H), 7.29 – 7.24 (m, 2H), 7.24 – 7.20 (m, 1H), 7.17 (dd, *J* = 2.5, 1.7 Hz, 1H), 6.92 (ddd, *J* = 8.2, 2.6, 0.9 Hz, 1H), 6.90 – 6.85 (m, 2H), 3.82 (s, 3H), 3.70 (s, 2H), 3.63 (s, 2H), 2.69 (s, 1H). HRMS (TOF, ES+), $C_{22}H_{21}D_3NO_2$ [M+H]+ calc. mass 337.1990, found 337.1995.

10 1-(3'-Bromo-[1,1'-biphenyl]-4-yl)-*N*-(4-methoxybenzyl)methanamine

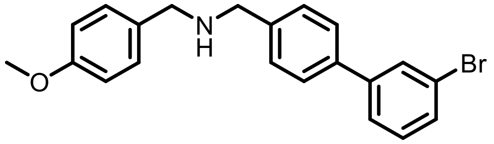

Followed General Procedure E with 3'-bromo-[1,1'-biphenyl]-4-carbaldehyde (50 mg, 0.19 mmol, 1 equiv) and (4-methoxyphenyl)methanamine (39.4 mg, 0.29 mmol, 1.5 equiv) to give the title compound as a tan gel after purification by column chromatography (0-80% EtOAc in hexanes) (43.5 mg, 59%). ^{1}H NMR (400 MHz, DMSO) δ 7.84 (t, *J* = 1.9 Hz, 1H), 7.67 (ddd, *J* = 7.8, 1.8, 1.1 Hz, 1H), 7.66 – 7.62 (m, 2H), 7.54 (ddd, *J* = 8.0, 2.0, 1.0 Hz, 1H), 7.46 – 7.38 (m, 3H), 7.29 – 7.24 (m, 2H), 6.91 – 6.85 (m, 2H), 3.73 (s, 3H), 3.72 (s, 2H), 3.64 (s, 2H). HRMS (TOF, ES+), $C_{21}H_{21}BrNO$ $[M+H]^+$ calc. mass 382.0801, found 382.0801.

11 1-(3'-(Methoxy-*d*$_3$)-[1,1'-biphenyl]-4-yl)-*N*-(4-methoxybenzyl)methanamine

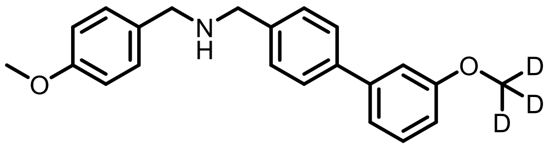

Followed General Procedure B with intermediate **10** (25 mg, 0.065 mmol, 1 equiv) and methanol-*d*$_4$ (0.027 mL, 0.65 mmol, 10 equiv) to give the title compound as a tan oil after purification by flash chromatography (0-50% EtOAc in hexanes) (11.7 mg, 53%). ^{1}H NMR (400 MHz, DMSO) δ 7.64 – 7.59 (m, 2H), 7.44 – 7.39 (m, 2H), 7.36 (t, *J* = 7.9 Hz, 1H), 7.29 – 7.24 (m, 2H), 7.23 – 7.19 (m, 1H), 7.17 (dd, *J* = 2.6, 1.6 Hz, 1H), 6.91 (ddd, *J* = 8.2, 2.6, 1.0 Hz, 1H), 6.90 – 6.86 (m, 2H), 3.73 (s, 3H), 3.69 (s, 2H), 3.63 (s, 2H), 2.69 (s, 1H). HRMS (TOF, ES+), $C_{22}H_{21}D_3NO_2$ $[M+H]^+$ calc. mass 337.1990, found 337.1994.

12 1-(3'-Bromo-[1,1'-biphenyl]-4-yl)-*N*-(4-bromobenzyl)methanamine

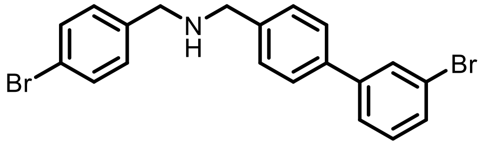

Followed General Procedure E with 4-bromobenzylamine (40 mg, 0.22 mmol, 1 equiv) and 4-(3-bromophenyl)benzaldehyde (84.2 mg, 0.323 mmol, 1.5 equiv) to give the title compound as a colorless oil after purification by flash column chromatography (0-70% EtOAc in hexanes) (77 mg, 83%). ^{1}H NMR (400 MHz, DMSO) δ 7.84 (t, *J* = 1.9 Hz, 1H), 7.71 – 7.60 (m, 3H), 7.55 – 7.47 (m, 3H), 7.48 – 7.39 (m, 3H), 7.36 – 7.29 (m, 2H), 3.71 (s, 2H), 3.67 (s, 2H). HRMS (TOF, ES+), $C_{20}H_{18}Br_2N$ $[M+H]^+$ calc. mass 431.9781, found 431.9782.

13 1-(3'-(Methoxy-*d*$_3$)-[1,1'-biphenyl]-4-yl)-*N*-(4-(methoxy-*d*$_3$)benzyl)methanamine

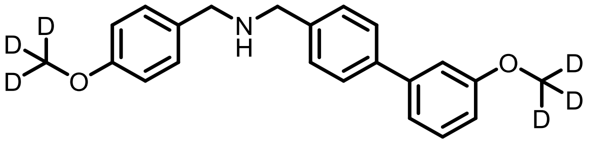

Followed General Procedure B with intermediate **12** (55 mg, 0.128 mmol, 1 equiv) and methanol-d_4 (0.104 mL, 2.55 mmol, 20 equiv) to give the title compound as a beige oil after purification by RP-HPLC (20-55% MeCN in 0.1% aqueous TFA solution) (32 mg, 74%). ^{1}H NMR (400 MHz, DMSO) δ 7.66 (d, *J* = 8.2 Hz, 2H), 7.46 (d, *J* = 8.1 Hz, 2H), 7.40 – 7.28 (m, 3H), 7.25 – 7.21 (m, 1H), 7.18 (t, *J* = 2.1 Hz, 1H), 6.97 – 6.87 (m, 3H), 3.84 (s, 2H), 3.78 (s, 2H). HRMS (TOF, ES+), $C_{22}H_{18}D_6NO_2$ $[M+H]^+$ calc. mass 340.2178, found 340.2183.

Table S1. Rat PK PBL Cassette Data for Compounds 5s, 9, 11, and 13[a]

Compound	Elim. $t_{1/2}$ (h)	MRT (h)	CL_p (mL/min/kg)	V_{ss} (L/kg)	AUC (h*ng/mL)	K_p
**5s**	1.56	0.99	17.7	1.06	189	1.81
**9**	1.56	1.02	25.4	1.55	131	2.15
**11**	1.54	0.81	26.2	1.27	127	1.94
**13**	1.59	1.00	26.4	1.58	126	2.06

a. See DMPK experimental section for further details.

Table S2. Thallium Flux and Manual Patch Clamp (MPC) Potency Data for Compounds 5s, 9, 11, and 13[a]

Compound	K_{ir}2.1 Thallium Flux Potency IC_{50}, µM (% Inhibition)	K_{ir}2.1 MPC IC_{50}, µM (Emax %)	$K_{ir}2.1/K_{ir}2.2$ Selectivity (MPC)	$K_{ir}2.1/K_{ir}2.3$ Selectivity (MPC)
**5s**	5.9 (101)	0.35 (100)	2.1 fold	2.5 fold
**9**	5.4 (100)	0.21 (99)	1.4 fold	1.8 fold
**11**	4.3 (100)	0.33 (99)	0.73 fold	1.3 fold
**13**	4.7 (100)	0.21 (99)	3.2 fold	2.4 fold

a. See thallium flux and manual patch clamp experimental sections for further details.

Thallium Flux Assay Experimental Conditions

The Tl^+ flux assays were conducted as previously described with modifications optimized for $K_{ir}2.1$ cell line as described below.[1]

Briefly, stable T-REx-HEK-293 cells expressing human $K_{ir}2.1$ were cultured in DMEM growth medium (Catalog #11965-092, Gibco, Billings, MT) containing 10% heat-inactivated FBS (Catalog #16140, Gibco), 100 U/mL PenStrep, 5 µg/mL Blasticidin S, and 250 µg/mL Hygromycin. The day prior to the experiment, cells at ~90% confluency were resuspended in plating media, which consisted of the same components as the growth media, except that heat-inactivated FBS was replaced with dialyzed FBS (Catalog #26400, Gibco). Tetracycline was added to the plating media to achieve a final concentration of 1 µg/ml to induce $hK_{ir}2.1$ expression. Cells were then counted and plated at 15,000 cells/well in black-walled, amine- or PDL- coated, 384-well plates at 20 µl/well (Corning, Corning, NY). On the day of experiment, the media was removed from the cell plate and replaced with 20 µl/well of Tl^+-sensitive dye loading solution containing 2.5 µg/ml Thallos-AM (Catalog #11000-050, ION Biosciences, San Marcos, TX) in assay buffer using a Multidrop Combi dispenser (Thermo Fisher Scientific, Walkham, MA). Cell plates loaded with dye solution were incubated for 1 hour at room temperature. Following incubation, the cell plate was washed with HBSS assay buffer containing 20 mM HEPES using a BioTek ELx 405 TS plate washer (Agilent, Santa Clara, CA), leaving 20 µl of assay buffer in each well. The cell plate was then transferred to a Panoptic kinetic imaging plate reader (WaveFront Biosciences, Franklin, TN) for imaging at 1 Hz, 482/35 nm excitation and 536/40 nm emission for 10 seconds. Following this, 20 µl/well of HBSS assay buffer containing test compounds at 2-fold over their final concentrations were added and imaging continued for another eight minutes, after which 10 µl/well of chloride-free Tl^+ stimulus solution was added (Catalog #11000-050, ION Biosciences). The final Tl_2SO_4 concentration in cell plate post-Tl^+ stimulus solution addition was 0.25 mM. Images were collected for an additional two minutes. A ten-point concentration series from 30 µM to 2 nM with a 1:3 dilution factor was generated using an Echo liquid handler 650 (Labcyte, San Jose, CA). Final DMSO concentration, 0.3% (*v/v*) in the assay was constant across all compound concentrations. The initial slopes of the Tl^+-induced fluorescence changes were fit to a four-parameter logistic equation using our in-house software Waveguide to determine potency and efficacy values. Efficacies are relative to a maximally effective concentration of our standard compound ML133.

The Tl^+ flux assays for $K_{ir}1.1$ and $K_{ir}4.1$ were conducted as previously described.[2]

1. Li, K.; McClenahan, S. J.; Han, C.; Bungard, J. D.; Rathnayake, U.; Boutaud, O.; Bauer, J. A.; Days, E. L.; Lindsley, C. W.; Shelton, E. L.; Denton, J. S. Discovery and Characterization of VU0542270, the First Selective Inhibitor of Vascular Kir6.1/SUR2B K_{ATP} Channels. *Mol. Pharmacol.* **2024**, *105*, 202-212.

2. McClenahan, S. J.; Kent, C. N.; Kharade, S. V.; Isaeva, E.; Williams, J. C.; Han, C.; Terker, A.; Gresham III, R.; Lazarenko, R. M.; Days, E. L.; Romaine, I. M.; Bauer, J. A.; Boutaud, O.; Sulikowski, G. A.; Harris, R.; Weaver, C. D.; Staruschenko, A.; Lindsley, C. W.; Denton, J. S. VU6036720: The First Potent and Selective In Vitro Inhibitor of Heteromeric Kir4.1/5.1 Inward Rectifier Potassium Channels. *Mol. Pharmacol.* **2022**, *101*, 357-370.

Manual Patch Claim Assay Experimental Conditions

Whole-cell patch-clamp electrophysiology was used to record $K_{ir}2.1$ currents from a stably transfected monoclonal T-Rex-HEK-293 cell line with a tetracycline-inducible promoter expressing human $K_{ir}2.1$, as described previously[1] cultured in a 5% CO_2 incubator at 37°C. $K_{ir}2.1$ expression was induced by overnight treatment with 1 µg/mL tetracycline. Cells were dissociated using 0.25% Trypsin-EDTA and seeded onto 12 mm circular coverslips (Electron Microscopy Sciences, PA, USA) for same-day whole-cell patch-clamp recordings. $K_{ir}2.1$ currents were recorded at room temperature using an AxoPatch 200B amplifier with Clampex 10.7 software (Molecular Devices, USA). The voltage-clamp protocol consisted of a 1-s step to -120 mV from a -80 mV holding potential, followed by a 2-s ramp to +40 mV, repeated every 10 s. Data were sampled at 5 kHz and filtered at 1 kHz. Patch pipettes, pulled from 1.5 mm OD thin-walled capillaries (Warner Instruments, MA, USA) using a Sutter P-1000 puller (Sutter Instrument, CA, USA), had resistances of 2–3 MΩ when filled with intracellular solution containing (mM): 135 KCl, 10 HEPES, 1 EGTA, 2 $MgCl_2$, 2 Na_2ATP (pH 7.35, adjusted with KOH). The extracellular solution contained (mM): 135 NaCl, 5 KCl, 2 $CaCl_2$, 1 $MgCl_2$, 5 Glucose, 10 HEPES, 10 Sucrose (pH 7.35, adjusted with NaOH). After establishing stable baseline $K_{ir}2.1$ currents, cells were perfused with compounds at concentrations of 0.03–30 µM until response saturation (within minutes). Recordings concluded with a 2 mM Ba^{2+} solution to isolate Ba^{2+}-sensitive currents, with compound responses expressed as fractions of this current. Current amplitudes at the -120 mV step were analyzed using Clampfit 11.2 (Molecular Devices). IC_{50} values were calculated by fitting the Hill equation to concentration-response curves (CRCs) using variable-slope nonlinear regression in GraphPad Prism 10.4 (GraphPad Software).

MPC experiments for $K_{ir}2.2$ and $K_{ir}2.3$ were conducted in an analogous fashion.[1]

1. Raphemot, R.; Swale, D. R.; Dadi, P. K.; Jacobson, D. A.; Cooper, P.; Wojtovich, A. P.; Banerjee, S.; Nichols, C. G.; Denton, J. S. Direct Activation of β-Cell K_{ATP} Channels with a Novel Xanthine Derivative. *Mol. Pharmacol.* **2014**, *85*, 858-865,

PK PBL Cassette Experimental Conditions

In-life phase

4 compounds plus one control were formulated as a solution in ethanol, PEG400, and DMSO (1:3:6 v/v, respectively) at a concentration of 1 mg/mL and administered as a single IV dose (1 mL/kg, 0.2 mg/kg per compound, total 1 mg/kg) to male, Sprague Dawley rats (n = 1) via injection into a surgically-implanted jugular vein catheter. Blood samples were collected serially from a surgically implanted carotid artery catheter in each animal over multiple post-administration time points (0.033, 0.117, 0.25, 0.5, 1, 2, 4, 7, and 24 hours) into chilled, K_2EDTA anticoagulant-fortified tubes and immediately placed on wet ice. The blood samples were then centrifuged (1700 rcf, 5 minutes, 4 ˚C) to obtain plasma samples, which were stored at -80 ˚C until analysis by LC-MS/MS.

For determination of the brain over plasma ratio (K_p), the same cassette of compounds plus control were formulated in ethanol, PEG400, and DMSO (1:3:6 v/v, respectively) and administered as a single IV dose (1 mL/kg, 0.2 mg/kg per compound, total 1 mg/kg) to male, Sprague Dawley rats (n = 1) via injection into a surgically-implanted jugular vein catheter. At 15 min post dosing, blood sample was collected terminally into chilled, K_2EDTA anticoagulant-fortified tube and immediately placed on wet ice. The blood sample was then centrifuged (1700 rcf, 5 minutes, 4 ˚C) to obtain plasma sample. At the same post-administration time point, whole brain sample was obtained by rapid dissection, rinsed with PBS, and immediately frozen in individual tissue collection box (dry ice). All brain and plasma samples were stored at -80 ˚C until analysis by LC-MS/MS.

Samples preparation for bioanalysis

Plasma samples from the in-life phase of the study were thawed at ambient temperature (benchtop), and then aliquots (20 µL per sample) were transferred to a 96-shallow-well (V-bottom) plate. Matrix-matched quality control (QC) samples and a standard curve of each analyte (1 mg/mL DMSO stock solution) were prepared in blank rat plasma (K_2EDTA-treated) via serial dilution and transferred (20 µL each) to the plate along with multiple blank plasma samples. MeCN (120 µL) containing IS (10 nM carbamazepine) was added to each well of the plate to precipitate protein. The plate was then centrifuged (4000 rcf, 5 minutes, ambient temperature), and resulting supernatants (60 µL each) were transferred to a new 96-shallow-well (V-bottom) plate containing an equal volume (60 µL per well) of water (Milli-Q purified). The plate was then sealed in preparation for LC-MS/MS analysis.

Preparation of brain samples was identical to that of plasma samples except for the following modifications. While thawing, brains were weighed (inside their collection boxes using a universal empty collection box tare weight) and then subjected to mechanical homogenization (Mini-BeadBeater™, BioSpec Products, Inc., Bartlesville, OK) in the presence of zirconia/silica beads (1.0 mm) and extraction buffer (isopropanol:water, 7:3, v/v; 3 mL per sample, corrected for post-quantitation). Homogenized brain samples were then centrifuged (4000 rcf, 5 minutes, ambient temperature), and 5 µL of the supernatant was diluted in 15 µL of blank plasma for quantification of the analyte. The plasma standard curve and QCs were used for compound quantitation in brain.

LC-MS/MS analysis

Prepared samples were injected (10 µL each) onto an AB Sciex Triple Quad 4500 mass spectrometer system with an Agilent 1260 Infinity II pump and autosampler. Mass spectrometer conditions are described in table 1 Quantitation of the analytes was performed via AB Sciex Multiquant software using the raw analyte:IS peak area ratios. The typical detection range was 0.5 ng/mL to ≥ 5,000 ng/mL utilizing a quadratic equation regression with $1/x^2$ weighting.

Correction for dilution of all brain samples (in extraction buffer and subsequently in blank plasma) was performed post-quantitation. The corrections for dilution in extraction buffer employed correction factors specific to each brain weight.

Table S3. LC-MS/MS Conditions

Injection volume	10 µL	
Mobile phase A	0.5% Formic Acid in Water	
Mobile phase B	0.5% Formic Acid in Acetonitrile	
Flowrate	0.5 mL/min	
Gradient	Time	% Mobile Phase B
	0.0	5
	0.2	5
	0.8	95
	1.5	95
	1.7	5
	2.7	Stop
Column	Fortis C18 (50 x 3.0 mm, 3 µm)	
Data collection and analysis software/version	Analyst v. 1.7.1	
Ionization mode	Positive Electrospray	
Collision gas (psi)	9	
Curtain gas (psi)	40	
GS1 (psi)	40	
GS2 (psi)	40	
Capillary voltage (V)	5500	
Source TurboIonSpray® temp. (°C)	500	
